# Extracellular Vesicle Measurements with Nanoparticle Tracking Analysis: A Different Appreciation of Up and Down Secretion

**DOI:** 10.3390/ijms23042310

**Published:** 2022-02-19

**Authors:** Clément Auger, Aude Brunel, Tiffany Darbas, Hussein Akil, Aurélie Perraud, Gaëlle Bégaud, Barbara Bessette, Niki Christou, Mireille Verdier

**Affiliations:** 1 UMR Inserm 1308, CAPTuR, Faculty of Medicine, University of Limoges, 2 rue du Dr. Marcland, 87025 Limoges, France; clement.auger@unilim.fr (C.A.); aude.brunel@unilim.fr (A.B.); tiffany.darbas@gmail.com (T.D.); aurelie.perraud@unilim.fr (A.P.); gaelle.begaud@unilim.fr (G.B.); barbara.bessette@unilim.fr (B.B.); christou.niki19@gmail.com (N.C.); 2 Service d’Oncologie, CHU of Limoges, 2 rue Martin Luther King, 87025 Limoges, France; 3 UMR CNRS 7276/INSERM U1262, Faculté de Médecine, Université de Limoges, 2 rue du Martin Luther King, 87025 Limoges, France; hussein.akil@unilim.fr; 4 Endocrine, General and Digestive Surgery Department, CHU of Limoges, 2 rue Martin Luther King, 87042 Limoges, France; 5 Laboratoire de Chimie Analytique, Faculté de Medecine & Pharmacie, 87025 Limoges, France

**Keywords:** extracellular vesicles, exosomes, nanoparticle tracking analysis (NTA), RAB27A, glioblastoma, colorectal cancer

## Abstract

As is the case with most eucaryotic cells, cancer cells are able to secrete extracellular vesicles (EVs) as a communication means towards their environment and surrounding cells. EVs are represented by microvesicles and smaller vesicles called exosomes, which are known for their involvement in cancer aggressiveness. The release of such EVs requires the intervention of trafficking-associated proteins, mostly represented by the RAB-GTPases family. In particular, RAB27A is known for its role in addressing EVs-to-be secreted towards the the plasma membrane. In this study, shRNAs targeting RAB27A were used in colorectal (CRC) and glioblastoma (GB) cell lines in order to alter EVs secretion. To study and monitor EVs secretion in cell lines’ supernatants, nanoparticle tracking analysis (NTA) was used through the NanoSight NS300 device. Since it appeared that NanoSight failed to detect the decrease in the EVs secretion, we performed another approach to drop EVs secretion (RAB27A-siRNA, indomethacin, Nexihnib20). Similar results were obtained i.e., no variation in EVs concentration. Conversely, NTA allowed us to monitor EVs up-secretion following rotenone treatment or hypoxia conditions. Therefore, our data seemed to point out the insufficiency of using only this technique for the assessment of EVs secretion decrease.

## 1. Introduction

Extracellular vesicles consist of heterogenous small vesicles bounded by portions of the plasma membrane and secreted by most eukaryotic cells, being healthy or pathological. They are classically divided into microvesicles, exosomes, and apoptotic bodies, the classification being mainly based on the biogenesis, size, and composition of the vesicle. Whereas microvesicles and apoptotic bodies directly bud from the plasma membranes, exosomes are products of the endosomal pathway and the multivesicular bodies [1]. Due to the variety of origins, the size range is between 30 and 1000 nm, exosomes being known to be the smallest (30–150 nm) [2]. The last ones are responsible for the transport of intracellular molecules, such as proteins, lipids, and nucleic acids, to the cell environment. The content of the vesicles is both the reflection of the donor cell’s unsorted composition and the result of a selection, in addition to non-specific exosomal constitutive proteins. Therefore, these vesicles perform functions in cell signaling, modifying the surrounding cells’ behavior. This capacity is particularly important in tumor development, in which exosomes have been described to be implied in cell proliferation regulation, angiogenesis, and metastasis [3]. This explains why there is an ever-increasing number of studies focusing on their role in oncogenesis.

Exosomes’ formation starts with the endosomal compartment. Early endosomes, generated by internal budding of the plasma membrane, progressively mature in multivesicular bodies (MVBs). Within these structures, invagination of their inner membrane generates intraluminal vesicles (ILVs), sized between 50 and 90 nm and containing parts of the cytosol. Finally, the MVBs fuse with the plasma membrane and release these vesicles, then called exosomes. All these steps are highly orchestrated and regulated, and the main role is played by the ESCRT (endosomal sorting complex required for transport) machinery. Four different complexes (ESCRT-0 to ESCRT-III) interplay in a sequential manner, along with accessory proteins, in order to drive the selection of participative molecules and the scission of the membrane. Nevertheless, an ESCRT-independent pathway has also been described, especially involving tetraspanins. At the end, depending on the efficiency of cargo sorting, life conditions, and the cells’ type, the composition of exosomes may vary.

When formed, these vesicles have to travel into the cell to be secreted. An important family of proteins involved in MVB trafficking is the RAB-GTPases family [1,4,5]. Several Rab proteins (5, 7, 11, and 35) mainly manage the intracellular movement along the microtubules, while RAB27A and RAB27B have been described to be crucial for exosome secretion. Both isoforms are responsible for the docking and fusion of MVBs on the plasma membrane, the last step before exosome release. In addition, it is also suspected that RAB27A endorses a role in controlling either the types of secreted EVs and/or their contents [6].

When working with such EVs, or more strictly with exosomes, one of the difficulties lies in the reproducibility and standardization of used techniques [7]. According to the EVs-studying scientific community, separation is predominantly realized using differential ultracentrifugations, using Théry’s protocole [8]. Subsequently, since the number and the content of EVs both reflect the cell of origin and influence the behavior of the receiver cell, these two parameters are crucial to investigate. Mainly Western blot, flow cytometry, electronic microscopy, surface plasma resonance (SPR), and nanoparticle tracking analysis (NTA) are used, all exhibiting advantages and limitations [2,9]. The last one is a light-scattering-based method that tracks the Brownian motion of each particle individually in order to determine the mean square displacement of individual particles [10]. NTA devices differ strongly in their hardware and software, affecting measuring results [11]. Moreover, in order to demonstrate the suitability of each experimental approach to measure the particle size and concentration of complex samples, testing reference materials mimicking the complexity of the biological samples as quality control prior to the measurements is strongly suggested [12]. Unfortunately, a metrology standard reference material that is truly representative of EVs is not yet available for measurements of particle size distribution and concentration [13].

In the present study, our initial aim was to evaluate the effects of exosome secretion inhibition in two tumoral models: glioblastoma and colorectal cancer. Indeed, in these two models, as in most cancers, tumor-derived exosomes act as messengers between tumor cells and the microenvironment and are prone to enhancing aggressiveness through this communication [14]. Our starting hypothesis was to evaluate the effects of an exosomal secretion alteration on cancer stem subpopulation. To reach this goal, we performed stable transfection of two representative glioblastoma and colorectal cancer cell lines with three different shRNAs targeting RAB27A. We chose to silence this protein expression because, among RAB-GTPases members, this one appears to be fundamental in exosome release [15]. Whereas transcriptomic and proteomic analysis confirmed this down-regulation, we were not able to detect any variation with NTA analysis. The same results were obtained using siRNAs targeting RAB27A and pharmacological inhibition of EVs release (Nexhinib20 or indomethacin), whereas when exosome secretion was forced (rotenone, hypoxia), the enhancement of EVs concentration was detected. Our work showed the weakness of using only one characterization method to assess EVs secretion decrease.

## 2. Results

**Figure 1:**
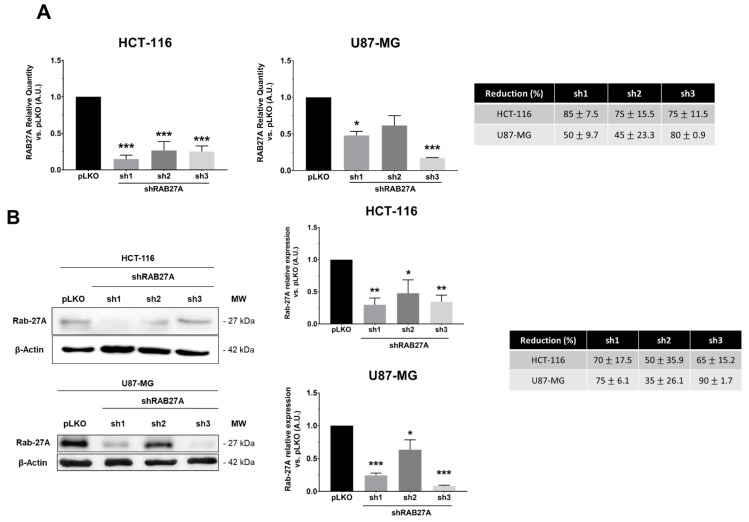

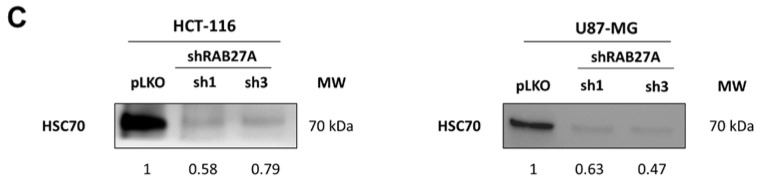
Validation of the shRAB27A models in both HCT-116 and U87-MG cell lines. (**A**)—Downregulation of RAB27A expression was evaluated by RT-qPCR in both pLKO (control) and shRAB27A cell lines. Whatever the cell line, sh1 and sh3 showed the strongest impairment in RAB27A expression compared to pLKO. The Mann–Whitney test was used for evaluation of the significance; * *p* < 0.05; *** *p* < 0.001 *n* = 6. (**B**)—Downregulation of RAB27A expression was evaluated by Western blot in HCT-116 and U87-MG models (left panels). The quantification of RAB27A expression (right panel) was consistent with RT-qPCR analysis. The Mann–Whitney test was used for evaluation of the significance; * *p* < 0.05; ** *p* < 0.01; *** *p* < 0.001; *n* = 6. (**C**)—Western blot analysis showed a decreased expression of HSC-70 in purified exosomes from both cell lines when RAB27A was down regulated; *n* = 3.

In order to study the impact of exosomes on cellular behaviors from glioblastoma (U87-MG cell line) or colorectal cancer (HCT-116 cell line), we silenced the expression of RAB27A. This GTPase protein, known to be a major actor in exosome release, was targeted by using stable transfection with three shRNAs against RAB27A (sh1, sh2, and sh3). As shown in Figure 1A,B, the different shRNA-RAB27As tested allowed a significant decrease in RAB27A expression, either at the transcriptomic or the proteomic level in both cancer models, i.e., HCT-116 and U87-MG, cultured in an exosome-free medium. Since we performed RAB27A silencing to decrease exosomes secretion (and thereby extend EVs secretion), we checked the expression of a well-known exosome marker: HSC70 (Figure 1C). As expected, Western blot analysis showed a decrease in the expression of this protein in exosomal extracts from both cell lines, indicating a decrease in exosomal secretion. Furthermore, when checking exosomal secretion in HCT-116 cell lines (pLKO and shRNA) using the on-bead flow cytometry approach, we observed a lower MFI for exosomes from RAB27A-silenced cells than for those from pLKO cells (respectively 3017 and 4963 for sh1 and sh3 and 12499 for pLKO; data not shown).

Populations sh1 and sh3 lead to the best down expression of RAB27A (over 70% of decreased expression). We chose these two populations for further experiments.

**Figure 2:**
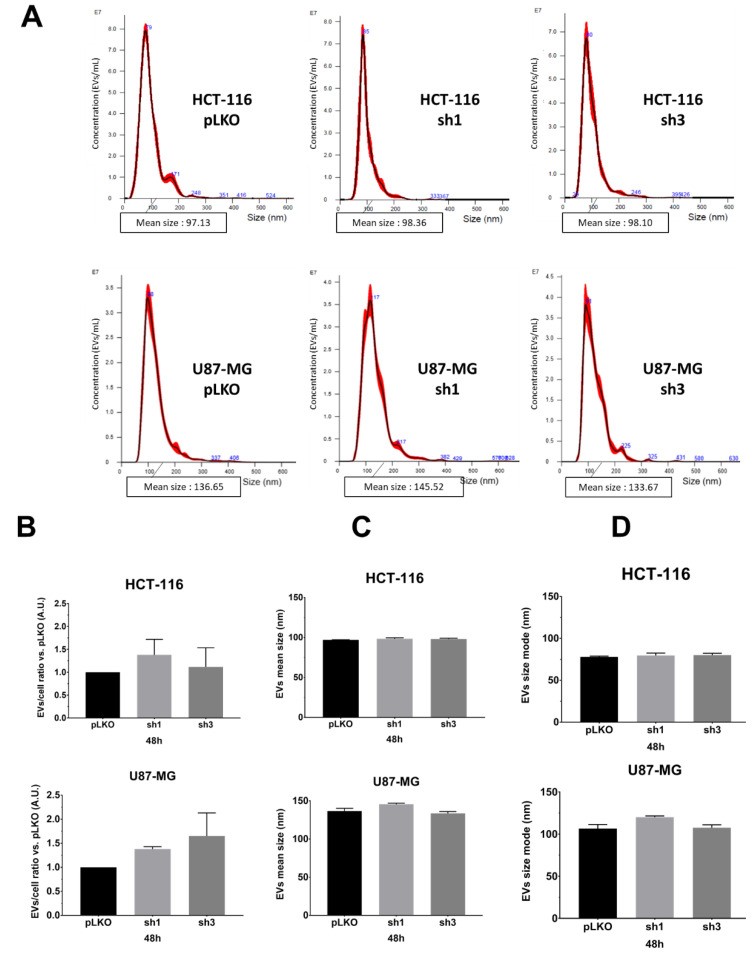

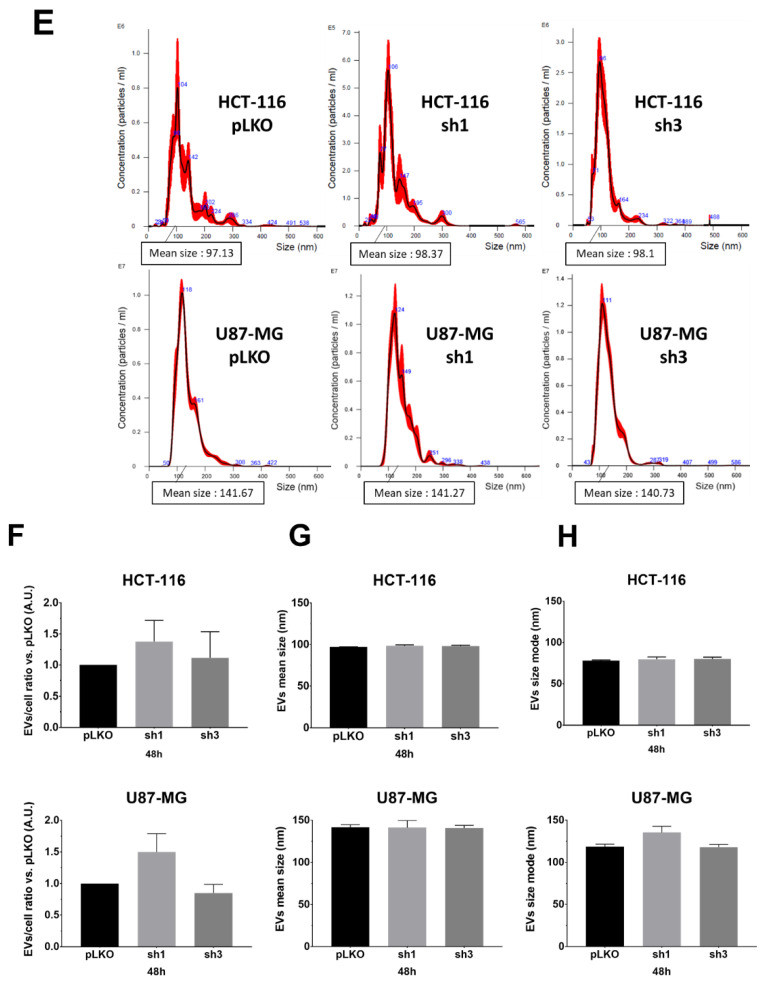
NTA analysis of extracellular vesicles from HCT-116 and U87-MG pLKO vs. shRAB27A. (**A**)—Evaluation of the size repartition of the EVs secreted by the HCT-116 (upper panel) and U87-MG (lower panel) pLKO and shRAB27A cell lines. The cells were cultured as mentioned in the material and methods, and the supernatants were directly analyzed using the Nanosight300. Most of the EVs population is comprised of between 50 and 180 nm, which is the size range commonly given to exosomes (*n* = 3). (**B**)—Concomitantly with the supernatant collection, cells were counted, and the EVs/cell ratio was calculated for each cell line. There is no difference concerning this ratio between pLKO and shRAB27A cell lines. (**C**)—EVs mean size (nm) from each cell line after 48 h of culture in EFM. (**D**)—EVs size mode (nm) from each cell line after 48 h of culture in EFM. There is no difference between the control and shRAB27A cell lines in both U87-MG and HCT-116, *n* = 3. (**E**–**H**)—The same analysis of EVs characteristics was performed at the same time (48 h) after the ultracentrifugation of the supernatant, according to Théry’s protocol (*n* = 3).

We monitored the EVs characteristics (EVs-per-cell ratio and size) using nanoparticle tracking analysis (NTA), using NanoSight NS300 (Malvern Pananalytical), following recommendations of the manufacturer. We opted to evaluate and compare EVs obtained either from the supernatant (only two centrifugation steps for the elimination of cells and debris), hereafter referred to as the crude supernatant (Figure 2A–D) or following the classical protocol, i.e., differential centrifugations [8] (Figure 2E–H). First, in order to optimize the detection of particles, we varied and tested the calibration settings on EVs suspension obtained after differential centrifugations of pLKO (scramble lentiviral vector) cells: camera level (12, 14, 16), detection threshold, PBS dilution rate (1/5, 1/10 depending on cell lines and exosome extraction methods), and acquisition time (60 s). After several assays, we fixed the following conditions for each sample: camera level 14, detection threshold 5, PBS dilution (1/10 when using crude supernatants, 1/5 when using ultracentrifugated supernatants), and five acquisitions of 60 sec each per sample. According to the manufacturer’s recommendations, we set the device to detect 50 to 120 particles/frame. Different experimental culture conditions (cell density; incubation time, i.e., 24 h, 48 h, 72 h) were also tested. Using such settings, we compared pLKO (scramble vector) and shRAB27A cells from the two different origins (HCT-116 and U87-MG) at two different culture times (48 h, Figure 2, and 72 h, Figure S1) and following two different collection techniques, i.e., crude supernatants (just with the first two steps of Théry’s protocol, 8) or after differential centrifugations. We did not observe any significant variation either in EVs concentration (particles/mL, Table 1) or in EVs number per cell (Figure 2B,F). The mean size and size mode (most represented size) of EVs also exhibited similar values, matching with classical EVs parameters, whatever the expression of RAB27A (Figure 2C,D,G,H and Figure S1). Furthermore, performing differential centrifugations did not allow us to improve NTA data, i.e., to detect a drop of EVs secretion (Table 1). This lack of difference between the two ways of obtaining EVs led us to continue the study with the analysis of EVs from crude supernatants.

Since apoptotic bodies are part of EVs family within the exosome size range (almost 100 nm), such results led us to evaluate cell death. Our hypothesis was that possible enhancement of apoptosis could compensate the impairment of exosomes due to targeting RAB27A. However, we did not detect any variation of cell death in shRNA-RAB27A cell lines either in HCT-116 or in U87-MG, which invalidated our hypothesis (data not shown).

**Figure 3:**
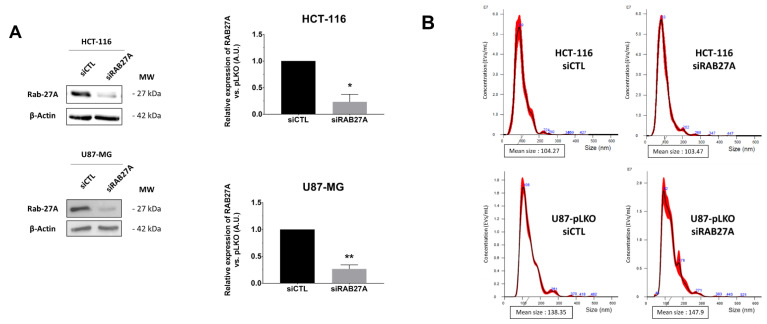

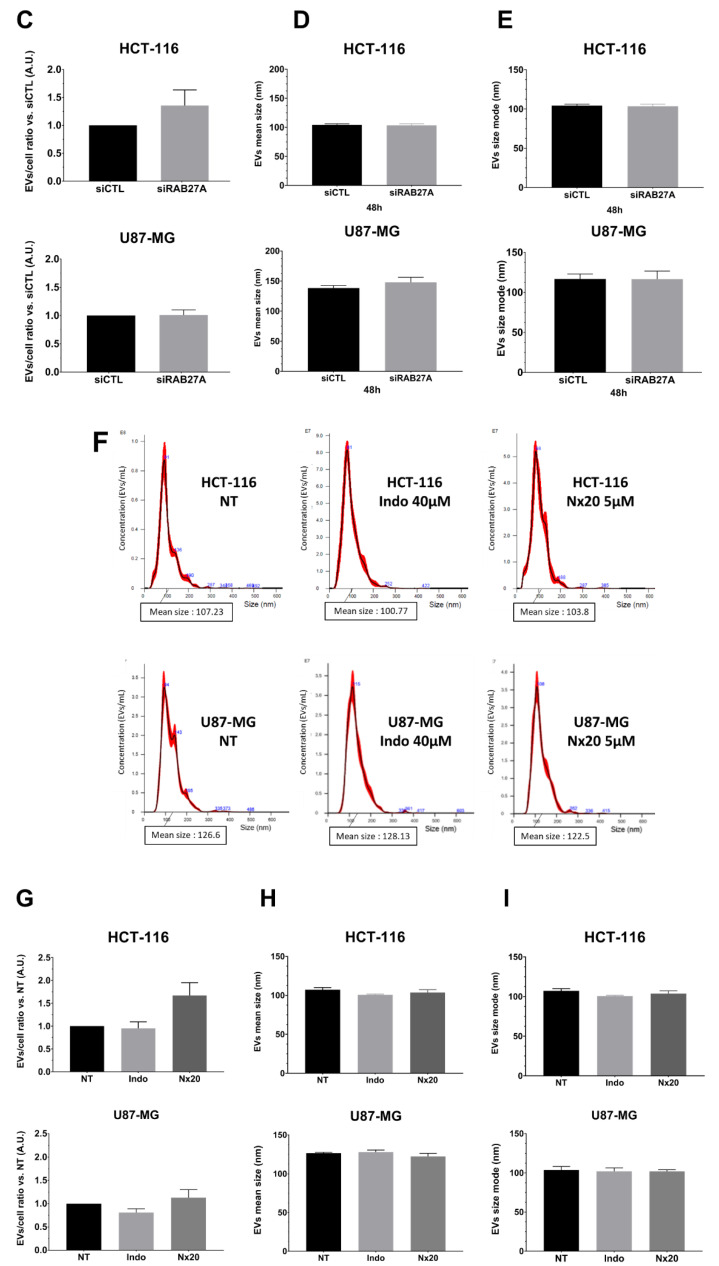
NTA analysis following different attempts for EVs secretion alteration in HCT-116 and U87-MG cell lines. (**A**)—Downregulation of RAB27A expression using siRAB27A was evaluated by Western lot (left panels). RAB27A expression quantification (right panels) in siCTL (control) and siRAB27A conditions in both HCT-116 and U87-MG cell lines (*n* = 3, *n* = 4, respectively). The Mann–Whitney test was performed in at least three independent experiments; * *p* < 0.05; ** *p* < 0.01. (**B**)—Evaluation of the size repartition of the EVs secreted by the HCT-116 (upper panel) and U87-MG (lower panel) pLKO transfected with siCTL or siRAB27A. (**C**)—EVs/cell ratio was calculated for each cell line. There is no difference concerning this ratio between pLKO siCTL and siRAB27A cell lines in both HCT-116 and U87-MG models (*n* = 3, *n* = 4, respectively). (**D**)—EVs mean size (nm) from each cell line after 48 h of culture in EFM. (**E**)—EVs size mode (nm) from each cell line after 48 h of culture in EFM. (**F**–**I**)—HCT-116 and U87-MG cell lines were treated in EFM with either indomethacin (*n* = 3, *n* = 4, respectively) or Nexinhib20 (*n* = 3, *n* = 6, respectively). (**G**)—EVs/cell ratio was calculated for each condition. There is no difference concerning this ratio between pLKO alone and pLKO treated with either indomethacin or Nx20 in both the HCT-116 and U87-MG models (*n* = 3 each). (**H**)—EVs mean size (nm) from each cell line after 48 h of culture in EFM. (**I**)—EVs size mode (nm) from each cell line after 48 h of culture in EFM.

Therefore, in a second step, in order to obtain a significant decrease in EVs secretion, we down-regulated expression of RAB27A by transient transfection with siRNA. After checking its efficiency on RAB27A expression (Figure 3A) and the duration of down expression (Figure S2A), we chose 48 h of siRNA treatment for further analysis because it was the most efficient time to decrease RAB27A expression using the siRNA approach. We thus evaluated EVs parameters in crude supernatants of 48 h cultured pLKO and siRNA cells (Figure 3B–E). Once again, NTA analysis of EVs in both cell lines did not show any significant decrease in EVs concentration per cell (Figure 3C), and the mean size and size mode were unchanged (Figure 3D,E).

In order to complete the genetic inhibition of RAB27A, we used a pharmacological approach. We chose to treat cells with Nexinhib20 or indomethacin since these two molecules are known to inhibit exosomes release in the range of working concentrations [16,17]. Using similar conditions, we checked that these treatments did not show any effect on RAB27A expression (Figure S2B). As for transfected cells with sh and siRNA targeting RAB27A, we evaluated EVs parameters through NTA analysis in crude supernatants of treated cells with one or another molecule (Figure 3F–I). As with sh- and si-RAB27A-transfected cells, we did not demonstrate any variation either in EVs concentration or in mean size and size mode after 48 h of treatment compared to pLKO untreated cells (Figure 3G–I). To summarize, these overall results, where we theoretically inhibited exosomes secretion by either genetic or pharmacological approach, did not reveal any impairment in EVs secretion when analyzed using NTA.

**Figure 4:**
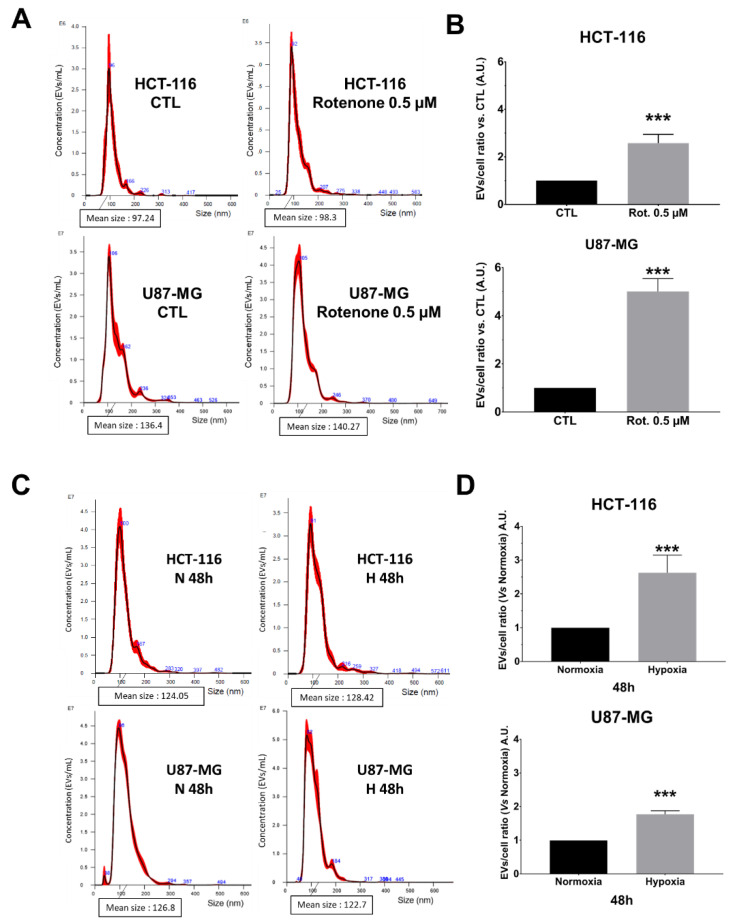
NTA analysis following EVs secretion increase attempts in HCT-116 and U87-MG cell lines. (**A**)—Evaluation of the size repartition of the EVs secreted by the HCT-116 (upper panel) and U87-MG (lower panel) untreated pLKO (CTL) or treated with 0.5µM of rotenone for 48 h. (**B**)—EVs/cell ratio was calculated for each condition in both HCT-116 and U87-MG cells (*n* = 3 each). The Mann–Whitney test was performed in three independent experiments; *p* < 0.05 was considered significant. (**C**)—EVs size range after 48 h under normoxia (N) or hypoxia (H) conditions was determined for both HCT-116 and U87-MG cells. (**D**)—EVs/cell ratio was calculated for each cell line in hypoxia compared to normoxia conditions. The Mann–Whitney test was performed in three independent experiments; *** *p* < 0.001.

Considering these data, we wondered if NTA allowed for monitoring EVs secretion’s enhancement. For this purpose, we either treated both cell lines with 0.5 µM of rotenone [18] or we cultured them under physic hypoxia (1% O_2_) for 48 h vs. normoxia (20% O_2_) conditions [19,20] (Figure 4). The EVs secretion in crude supernatants was significantly enhanced in rotenone-treated pLKO cells for both HCT-116 and U87-MG (Figure 4A,B; *** *p* < 0.001), without modification of the mean size and size mode (Figure S3A,B). Concerning hypoxic conditions, the NTA analysis showed that both cell lines produced significantly more EVs after 48 h of 1% O_2_ culture (Figure 4C,D; *** *p* < 0.001). The mean size and the size mode were not affected (Figure S3C,D). Such enhancement of EVs secretion started to appear straight after 24 h of hypoxia, even if it reached significant values only for U87-MG (Figure S4).

**Figure 5:**
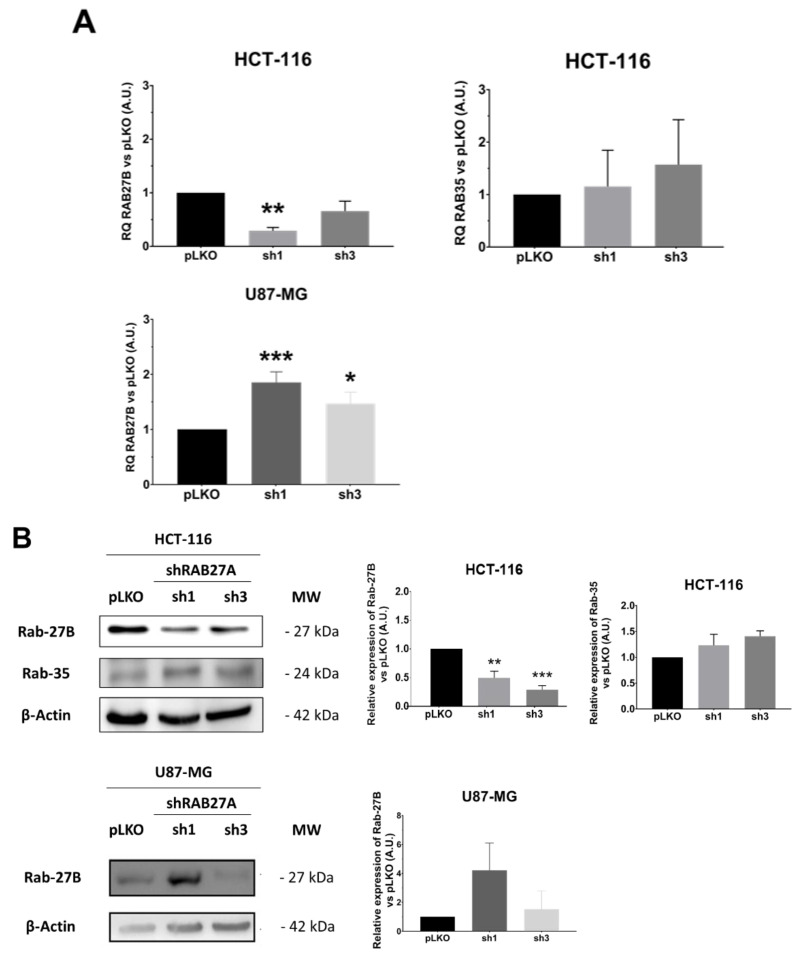

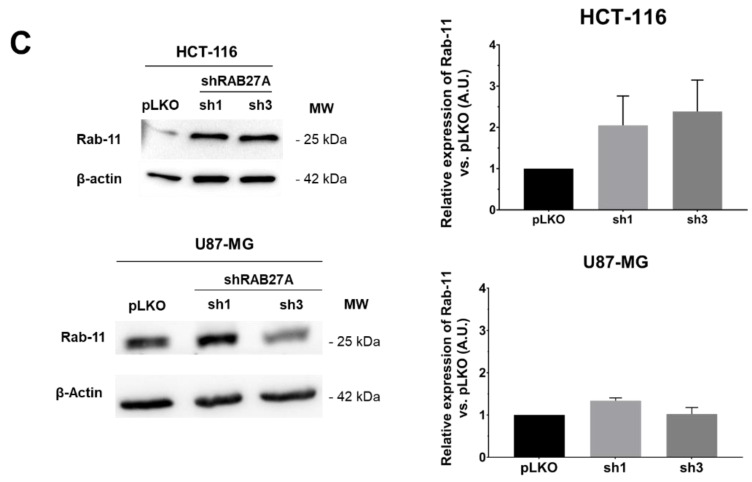
Analysis of the expression of other RAB-GTPases. (**A**)—RAB27B and RAB35 expressions were analyzed by RT-qPCR assays in both HCT-116 and U87-MG cell lines. RAB35 was not detected in the U87-MG cell line. The Mann–Whitney test was performed on three independent experiments; * *p* < 0.05; ** *p* < 0.01; *** *p* < 0.001. (**B**)—RAB27B and RAB35 expressions were assessed by Western blot in both HCT-116 and U87-MG cell lines. RAB35 failed to be detected in U87-MG. The Mann–Whitney test was performed in three independent experiments; ** *p* < 0.01; *** *p* < 0.001. (**C**)—RAB11 expression was analyzed by Western blot in both HCT-116 and U87-MG cell lines. The Mann–Whitney test was performed on three independent experiments.

Considering these data, i.e., EVs secretion enhancement detectable using NTA, whereas it did not seem to be the case for the decrease, we wondered if the cells were able or not to compensate for the RAB27A deficit. Indeed, this protein is not the only one implicated in the EVs secretion. Several other RAB family members, the foremost being RAB27B, could endorse this role [4,5,15]. Therefore, we evaluated the expression of three RAB members mainly involved in EVs secretion: RAB27B, RAB35, and RAB11 (Figure 5). The two studied cell lines seem to regulate in a divergent way the expression of RAB27B (Figure 5A). Indeed, whereas the CRC cell line HCT-116 exhibited a significant decrease in RAB27B along with shRAB27A transfection, ruling out any compensation by this isoform, in the GB cell line U87-MG, we observed an enhancement of RAB27B expression, especially at transcriptional level in the sh1 population, supporting a possible relay process. On the other hand, neither RAB35 (absent at the transcriptional and protein levels in the GB cell line) nor RAB11 expressions seemed to be affected by shRAB27A (Figure 5B,C).

In conclusion, even if results obtained with other RAB proteins seem to point to explanatory tracks, number-weighted concentration measurements with NTA are not convincing when it comes to showing a drop of EVs secretion.

## 3. Discussion

A main area of our research focusses on parameters of aggressiveness in solid tumors. In the past years, extracellular vesicles and, more precisely, exosomes, have increasingly been shown as important in such processes [3,14,21]. It is now accepted that they act as modulators o tumoral environments, allowing immune-regulation, angiogenesis, or stem cell education [22]. The initial aim of our work was to obtain tumoral cells unable to generate exosomes in order to analyze their behavior, especially concerning aggressiveness properties. We chose to alter RAB27A expression due to its encoded protein that is commonly addressed because of its role in exosomes secretion [6,15], which we checked by HSC-70 Western blot analysis. Indeed, it has been shown that both RAB27 A and B act in exosomes’ secretion by docking multivesicular bodies (known as the source of exosomes) to the the plasma membrane [23], which is recovered in several cancer cell types [24,25]. The quantification of EVs we performed using nanoparticle tracking analysis, before or after ultracentrifugation, failed to exhibit either reduced number or modified particle size, despite our having implemented several reduction approaches (i.e., genetic or pharmacological) in two different cellular models. Such results led to various questions.

Concerning EVs secretion reduction by the means of sh or si RNA, we cannot doubt that we reached the target, with 80–85% of extinction (as shown by transcriptomic and protein analysis). With a similar extinction rate, Zheng et al. [26] showed that NTA was performant to detect exosomes secretion decrease. However, the intra-assay variability could be substantially reduced by using an antibody-specific fluorescent label [27]. With the fluorescent filter in place, they were able to visualize a larger concentration of particles sized between 20 and 100 nm compared with the light scatter mode. This may be due to the intense light scatter from larger particles interfering with the accurate and reproducible measurement of smaller particles [27]. In our study, we also chose to reduce EVs secretion using pharmacological drugs (indomethacin and nexinhib20), which do not target RAB27A. Indomethacin is a cyclooxygenase inhibitor belonging to the non-steroidal anti-inflammatory drugs family. It is also known to reduce the expression of ABCA3, a lipid ABC transporter involved in exosome secretion [17,28,29]. Nexinhib20 is commonly used to prevent neutrophiles degranulation [16,30], and by extension, it is used to inhibit EVs secretion, which is a related process. However, once again, using either one or the other drug, NTA did not allow us to detect a drop in EVs’ secretion, maybe due to the lack of sensitivity of the technique.

We therefore took the opposite approach by forcing the cells to secrete more EVs. A previous study [31] showed that rotenone induces the formation and release of exosomes. Rotenone is a pea plant-derived molecule and is known to inhibit mitochondrial complex-I activity, leading to ROS and oxidative damage. Another way to induce EVs over-secretion was achieved by hypoxia [20,32,33]. Commonly, cells respond to oxygen privation through several mechanisms, such as EVs secretion leading to hypoxia tolerance [19]. This includes angiogenesis, autophagy, and EVs release, allowing specific communication with neighbor cells [34]. In our working conditions (1% O_2_ during 48 h; 0.5 µM of rotenone), we effectively detected an enhancement of EVs secretion in both cellular models by using NTA.

Therefore, at this time, the following question is raised: why did NTA allow us to detect an increase in EVs secretion, whereas it did not for a decrease? Basically, such a device was not developed for a quantitative use. According to the manufacturer, NanoSight, which functions according to NTA, was initially designed for the characterization of nanoparticles, not for EVs monitoring. Repeatability, reproducibility, and the particle concentration overestimation issues observed in the work of Vestad et al. [10] may be linked to the subjective choice of some measurement setting that must be carefully fine-tuned, depending on the particle size and on their optical properties. Previously, the best choice to check suitability and experimental performances was to use polydisperse mixtures of polymeric particles, such as polystyrene, despite their being very different in their chemical and physical properties compared with EVs [27]. The findings of Usfoor et al. [35] demonstrated the overestimation of particle concentration and that an approach of swift correction of the results of concentration measurements received for samples is suggested.

These studies highlight the general need to develop and validate new reference materials with similar physical and biochemical properties to EVs to standardize EVs measurements between instruments and laboratories [36]. In our study, although using the nominal concentration of nanoparticles is difficult, we can interpret the results obtained by simple comparison. Activation of EVs secretion, either by rotenone or hypoxia, leads to an increase in the amount of EVs of the same kind if we consider the EVs secreted by pLKO as an internal standard. These EV’s have comparable Brownian motion and refractive indices, allowing the NTA to be used as a tool to measure this increase. On the other hand, the inhibition of secretion did not appear to result in a decrease in EVs of the same kind. This population, although having a comparable size distribution and close nominal concentration, is probably constitutively different, understanding a population whose density or area distribution differs but has an identical signal, size, and concentration. This result opens new perspectives for the use of the NTA measurements. Indeed, it would then be advisable to extract from the NTA results the information of volume, thus of density as well as of surface occupied by each analyzed particle. The correlograms that could be obtained would allow a different EVs distribution analysis more likely to identify the biological phenomenon at stake.

Furthermore, considering that EVs secretion is a survival process for almost all tumoral cells, another explanation for not detecting EVs drop would be the implementation of another process to offset the loss of EVs. Indeed, other members of the RAB-GTPase family are able to endorse a function promoting EVs secretion instead of RAB27A [4,5,15]. When assessing the expression of RAB27B, RAB11 and RAB35, which may be the main potential actors of such a compensation, the only protein exhibiting an enhancement was RAB27B in the glioblastoma model. 

## 4. Materials and Methods

### 4.1. Cell Lines and Culture

U87-MG and HCT-116 cells were used as classical glioblastoma and colorectal cancer models, respectively. Both cell lines were provided by ATCC. U87-MG cells were cultured in MEM (Gibco, Life Technologies, Cergy-Pontoise, France) supplemented with 10% FBS, 1% NEAA, 1% sodium pyruvate, 0.75 g of sodium bicarbonate, and 1µg/mL of puromycin. HCT-116 cells were cultured in McCoy’s 5A Medium with L-glutamine (#BE12-688EF, Lonza, Levallois-Perret, France) supplemented with 10% FBS, 100 IU/mL of penicillin, and 100 mg/mL of streptomycin. In order to obtain stable down expression of RAB27A, both cell lines were infected with virions containing either three different shRNAs targeting RAB27A, named sh1, sh2, sh3, or a scramble vector as a control (pLKO cell lines). Polybrene was used at a final concentration of 8 µg/mL to help virions entering the cells. For the selection of plasmid-integrated cells, puromycin was used at a working concentration of 5µg/mL and 30µg/mL for U87-MG and HCT-116 cell lines, respectively. For each experiment, cells were seeded at a density of 10,000 cells/cm^2^ or 20,000 cells/cm^2^ for U87-MG and HCT-116, respectively, in anEVs-free medium (EFM) for different durations (24, 48, or 72 h). Cells were cultured either in normoxia (N; 5% CO_2_, 20 %O_2_) or in hypoxia (H; 5% CO_2_, 1% O_2_) at 37°C at a hypoxia workstation (InvivO2, Baker, Alliance Bio Expertise Guipry-Messac, France).

### 4.2. Cell Treatments

Nexinhib20 (Nx20; 5 µM, 2 h; Sigma-Aldrich, Saint Quentin Fallavier, France), initially described for the inhibition of neutrophils exocytosis [16], was used to inhibit the secretion of EVs by altering the RAB27A mode of action. Indeed, Nx20 should be an inhibitor of the interaction between RAB27A and its effector JFC1 (synaptotagmin-like protein 1, 16).

Indomethacin (Indo; 40 µM, 48 h; Sigma-Aldrich, Saint Quentin Fallavier, France) was used to interfere with the EVs secretion process by inhibiting the ABC transporter A3 (ABCA3, 17). Rotenone (Rot; 0.5 µM, 48 h; Sigma-Aldrich, Saint Quentin Fallavier, France) was used to increase the secretion of EVs by inducing stress within the mitochondrial respiratory chain [18,31].

Both U87-MG and HCT-116 pLKO cell lines were also treated with an siRNA-targeting RAB27A for 48 h (MISSION esiRNA targeting human RAB27A, Sigma-Aldrich, Saint Quentin Fallavier, France) to compare transient and stable downregulations.

For each experiment, cells were seeded in a complete medium for one night. The next day, the medium was changed for EFM at the time of the different treatments. The supernatants and the cells were collected 48 h following the treatments. Cells were counted, and cell viability was evaluated using a trypan blue exclusion assay.

### 4.3. EVs Isolation

EVs were isolated following an adaptation of Théry et al.’s protocol [8] using differential ultracentrifugation. First, the supernatants were centrifuged at 300× *g* for 10 min in order to eliminate the remaining cells. Then, a centrifugation at 2000× *g* for 10 min was achieved to pellet dead cells (crude supernatant). A 10,000× *g* centrifugation for 40 min was realized, and the supernatants were filtered using a 0.2 µm filter. A first ultracentrifugation at 120,000× *g* for 70 min was performed to obtain pellet containing EVs, which were washed in PBS before the second and last 70 min ultracentrifugation at 120,000× *g*. The pelleted and washed EVs were then resuspended either in PBS for nanoparticle tracking analysis, or in a cell lysis buffer for Western blot analysis.

Concerning NTA, the supernatants were analyzed either after the protocol described below or after only the 300× *g* and 2000× *g* centrifugations (named crude supernatant).

### 4.4. Western Blot Analysis

U87-MG cells were lysed using a cell lysis bBuffer (Cell Signaling Technology, Ozyme, Saint-Cyr-l’Ecole, France) for 30 min on ice. Then, a mechanical lysis was realized using 300 µL syringes and centrifuged for 20 min at 17,000× *g*. HCT-116 cells were lysed using a homemade RIPA buffer for 20 min on ice, then sonicated for 20 s (one pulse every 2 s) and centrifugated at 18,000× *g* for 30 min. In each case, supernatants were collected, and protein concentration was evaluated using a Bradford protein concentration assay (BioRad, Marnes-La-Coquette, France). SDS-PAGE was achieved on 30 µg of proteins for cell lysate or 10 µg for exosomes, and proteins were transferred on PVDF membranes (GE Healthcare, Tremblay-en-France, France). Membranes were incubated for 1 h in blocking solution (5% BSA or 5% nonfat dry milk in 1X TBS-0.1% Tween-20) and were then exposed to specific primary antibodies (anti-RAB27A (D7V6B), Cell Signaling #95394; anti-RAB27B, Cell Signaling #44813; anti RAB11 (D4F5 XP), Cell Signaling #5589; anti-RAB5, Cell Signaling #9690 (Cell Signaling Technology, Ozyme, Saint-Cyr-l’Ecole, France); anti-HSC-70, Santa Cruz sc-7298; anti-β actin, Santa Cruz sc-47778 (Santa-Cruz, Heidelberg, Germany) in a blocking solution overnight at 4 °C. Membranes were washed three times in TBS-0.1% Tween-20 before incubation during 1 h in a blocking solution at room temperature with the horseradish peroxidase-conjugated secondary antibody targeting mouse or rabbit immunoglobulins (Dako Cytomation, Les Ulis, France) diluted at 1:1000. Membranes were washed two times in TBS-0.1% Tween-20, then once in TBS before revelation using a G-Box (Syngene, Fisher Scientific, Illkirch, France) and the GeneSys application suite.

### 4.5. RT-qPCR Analysis

RNA was extracted from cells and isolated using an RNeasy Mini Kit (# 74106; QIAGEN, Hilden, Germany) according to the manufacturer’s protocol. After quantification using a NanoDrop 2000 (Thermo Scientific, Illkirch, France), 2 μg of total RNA were reverse transcribed with a high-capacity cDNA Reverse Transcription Kit (Applied Biosystems, Foster City, CA, USA). The qPCRs were achieved on 100 ng of cDNA using Premix Ex Taq (#RR39WR, TaKaRa, Saint Germain-en-Laye, France) on a QuantStudio 3 real-time thermal cycler (Applied Biosystems, Fisher Scientific, Illkirch, France) with TaqMan probes for each reaction. Probes used for quantitative RT-qPCR are listed in Table 2. Reactions were performed in triplicate from each biological replicate. Relative gene expression was quantified using glyceraldehyde-3-phosphate dehydrogenase (GAPDH), ubiquitin C (UBC), and hypoxanthine phosphoribosyltransferase 1 (HPRT1) as house-keeping genes. The 2^−ΔΔCt^ quantitative method was used to normalize expression of the reference gene and to calculate the relative quantification (RQ) levels of target genes.

### 4.6. Nanoparticle Tracking Analysis

NTA was performed using NanoSight NS300 (Malvern Panalytical Ltd., Malvern, UK) with specific parameters according to the manufacturer’s user manual (NanoSight NS300 User Manual, MAN0541-01-EN-00, 2017). Captures and analysis were achieved by using the built-in NanoSight Software NTA3.3.301 (Malvern Panalytical Ltd., Malvern, UK). The camera level was set at 14 as all particles were visible at this level without signal saturation, and the detection threshold was fixed at 5 to include most of the observed particles while excluding indistinct ones. Samples were diluted in PBS to a final volume of 1 mL, and their concentration was adjusted by observing a particles/frame rate of around 50 (30–100 particles/frame). For each measurement, five consecutive 60-s videos were recorded under the following conditions: cell temperature—25 °C, syringe speed—22 µL/s (100 a.u.). Particles (EVs) were detected using a 488 nm laser (blue), and a scientific CMOS camera. Among the information given by the software, the following were studied: mean size, mode (i.e., the most represented EVs size population), and particles/mL. Using the last one and the number of cells collected at the different incubation times, we were able to calculate an EVs/cell ratio for each run of each condition.

### 4.7. Statistical Analysis

Statistical analyses were performed using Past 3 software (National History Museum, University of Oslo). The Mann–Whitney test was used for multiple comparisons. At least three independent biological replicates were achieved for each experiment. The *p*-values of less than 0.05 were considered significant.

## 5. Conclusions

The limitations in using NTA for EVs quantification are regularly underlined. For example, Vestad et al. [10] reported intra- and inter- assay variations when same EVs samples were analyzed in different laboratories. Similar observations were also evidenced by other teams [9,37]. According to these authors, standardization is needed when using NTA to ensure proper quantification. Therefore, the results we obtained in our experimental conditions seem in accordance with previous observations, and we recommend remaining careful when monitoring EVs secretion using NTA. Additionally, EVs secretion, being such a complex pathway containing so many actors, seems to be tricky to target efficiently. Indeed, other proteins are able to take over the role of the downregulated protein, as shown in the glioblastoma cell line.

## Figures and Tables

**Table 1:** EVs NTA-estimated concentration for each condition in each cell line according to the different culture times (UC: Ultracentrifugation).

EVs/mL			pLKO	sh1	sh3
HCT116	48 h	Crude	4.46 × 10^9^	4.01 × 10^9^	4.36 × 10^9^
		UC	4.63 × 10^7^	3.20 × 10^7^	1.41 × 10^8^
	72h	Crude	1.58 × 10^9^	1.64 × 10^9^	1.85 × 10^9^
		UC 72 h	5.21 × 10^7^	1.26 × 10^8^	6.58 × 10^7^
U87	48 h	Crude	1.96 × 10^9^	2.37 × 10^9^	1.99 × 10^9^
		UC	6.22 × 10^8^	7.25 × 10^8^	7.87 × 10^8^
	72h	Crude	2.35 × 10^9^	3.01 × 10^9^	2.87 × 10^9^
		UC	7.05 × 10^8^	1.01 × 10^9^	6.49 × 10^8^

**Table 2:** References of probes used for quantitative RT-qPCR.

Gene	Supplier	Reference
GAPDH (GAPDH)	ThermoFisher Scientific	Hs02758991_g1
UBC (UBC)	ThermoFisher Scientific	Hs00824723_m1
HPRT1 (HPRT1)	ThermoFisher Scientific	Hs02800695_m1
RAB27A (RAB27A)	ThermoFisher Scientific	Hs00608302_m1
RAB27B (RAB27B)	ThermoFisher Scientific	Hs00188156_m1
RAB35 (RAB35)	ThermoFisher Scientific	Hs00199284_m1

## Data Availability

Not applicable.

## Supplementary Materials

The following supporting information can be downloaded at: https://www.mdpi.com/article/10.3390/ijms23042310/s1.
