# Supplementary material for: Extracellular Vesicle Measurements with Nanoparticle Tracking Analysis: A Different Appreciation of Up and Down Secretion
Source: Int J Mol Sci. 2022 Feb 19;23(4):2310. doi: 10.3390/ijms23042310 (PMC8875573; doi:10.3390/ijms23042310)
Supplement: Supplementary file 1 [file ijms-23-02310-s001.zip › ijms-1543152-supplementary.pdf]

International Journal of *Molecular Sciences*

MDPI

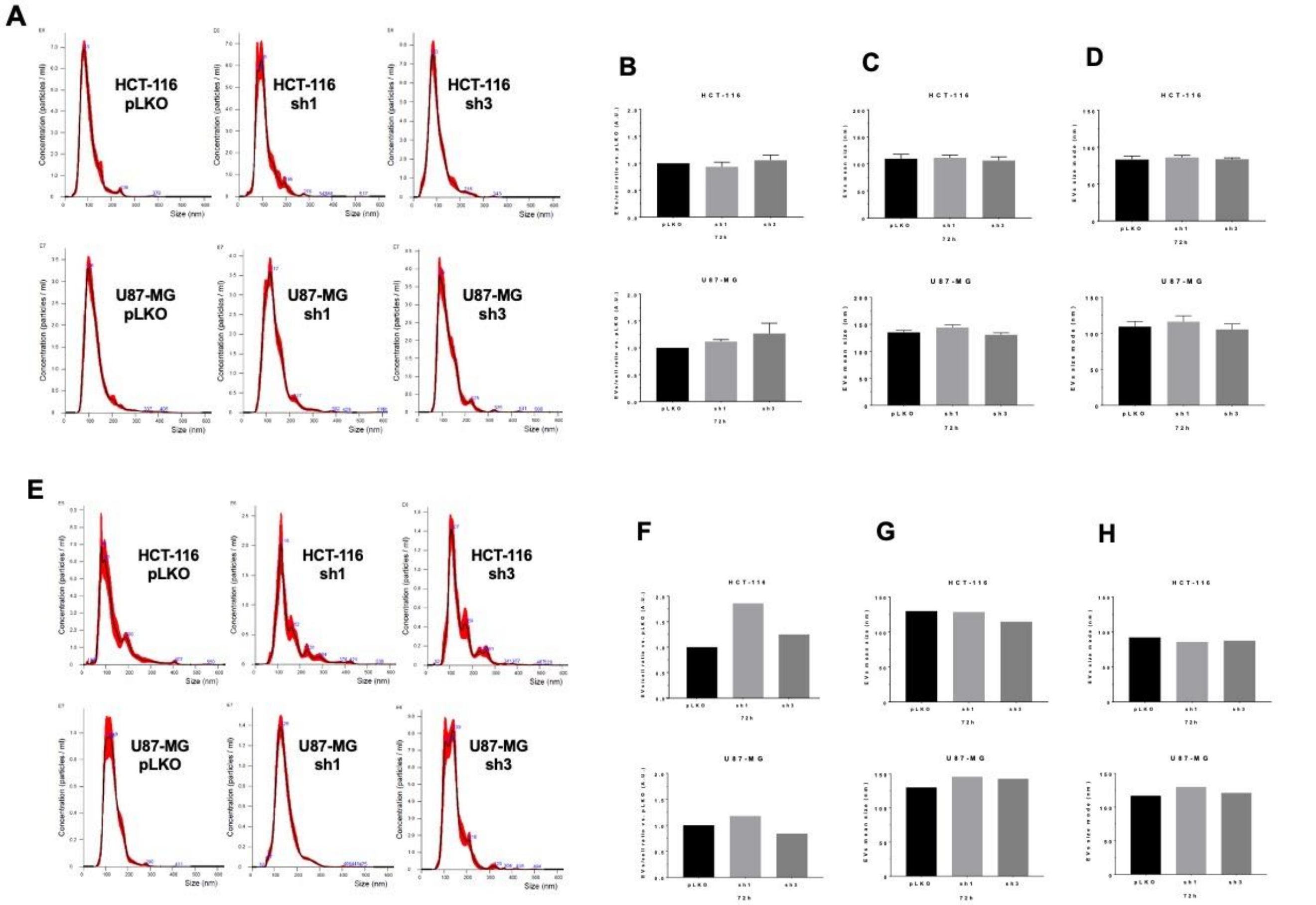

Figure S1. Evaluation of the characteristics of the EVs secreted by the HCT-116 (upper panel) and U87-MG (lower panel) pLKO and shRAB27A cell lines after 72h of culture either in crude supernatant (**A, B, C, D**) or after performing differential ultracentrifugations (**E, F, G, H**). There is no difference between the control and shRAB27A cell lines, in both U87-MG and HCT-116.

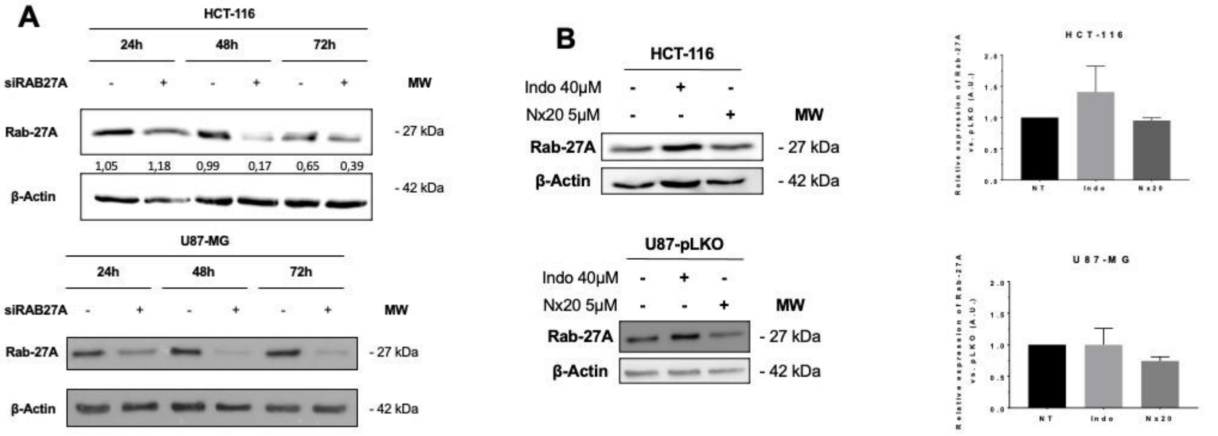

Figure S2. A – Downregulation of Rab-27A expression was evaluated by Western Blot in HCT-116 (upper panel) and U87-MG (lower panel) cell lines at different times of siRAB27A treatment. **B** – Rab-27A expression was evaluated by performing Western Blot analysis in indomethacin or Nexinhib20 treated cells (left panels). Rab-27A expression quantification (right panels) in pLKO cell lines (control) and in the different treatment conditions. This expression is not affected by any of the

treatment conditions. Mann-Whitney test was performed on three independent experiments; p<0.05 was considered as significant.

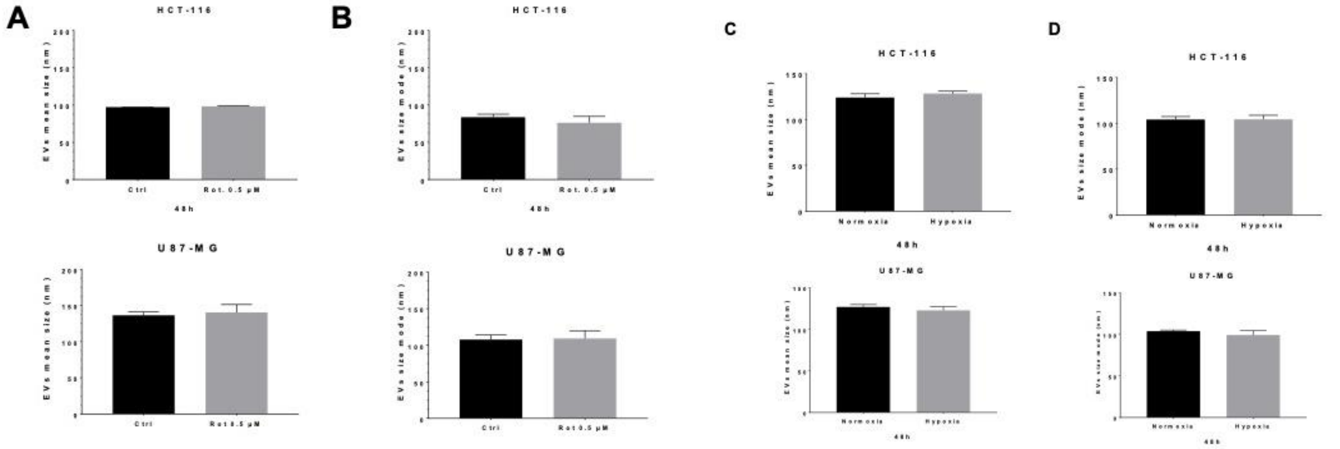

Figure S3. A & C – EVs mean size (nm) from each cell line after respectively either rotenone treatment or hypoxia conditions in EFM. **B & D** – EVs size mode (nm) from each cell line after respectively either rotenone treatment or hypoxia conditions in EFM. There is no difference between the different conditions i.e. treated or not with rotenone and normoxic or hypoxic conditions, in both U87-MG and HCT-116. Mann-Whitney test was performed on three independent experiments; p<0.05 was considered as significant.

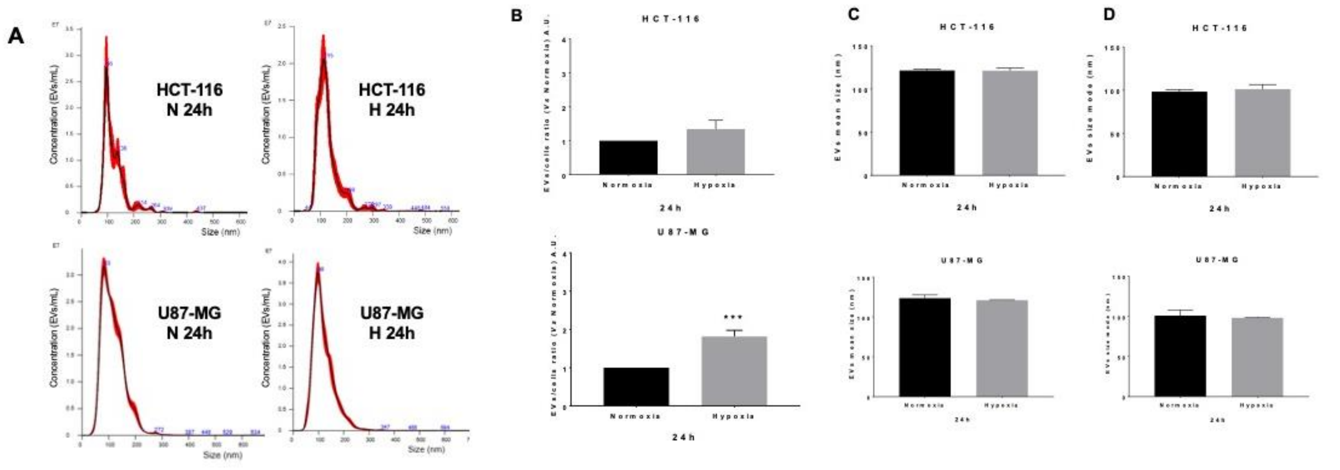

Figure S4. Evaluation of the characteristics of the EVs secreted by HCT-116 (upper panel) and U87-MG (lower panel) when cultured during 24h in hypoxic conditions. **A** – EVs size range after 24h under normoxia or hypoxia conditions was determined for both HCT-116 and U87-MG cells. **B** – EVs/cell ratio was calculated for each cell line in hypoxia compared to normoxia condition. Mann-Whitney test was performed on three independent experiments; p<0.05 was considered as significant. **C** – EVs mean size (nm) from each cell after 24h of culture in EFM. **D** – EVs size mode (nm) from each cell line after 24h of culture in EFM. Mann-Whitney test was performed on three independent experiments; p<0.05 was considered as significant.
