# Supplementary figures and images for: Extracellular Vesicle Measurements with Nanoparticle Tracking Analysis: A Different Appreciation of Up and Down Secretion
Source: Int J Mol Sci. 2022 Feb 19;23(4):2310. doi: 10.3390/ijms23042310 (PMC8875573; doi:10.3390/ijms23042310)

**A**

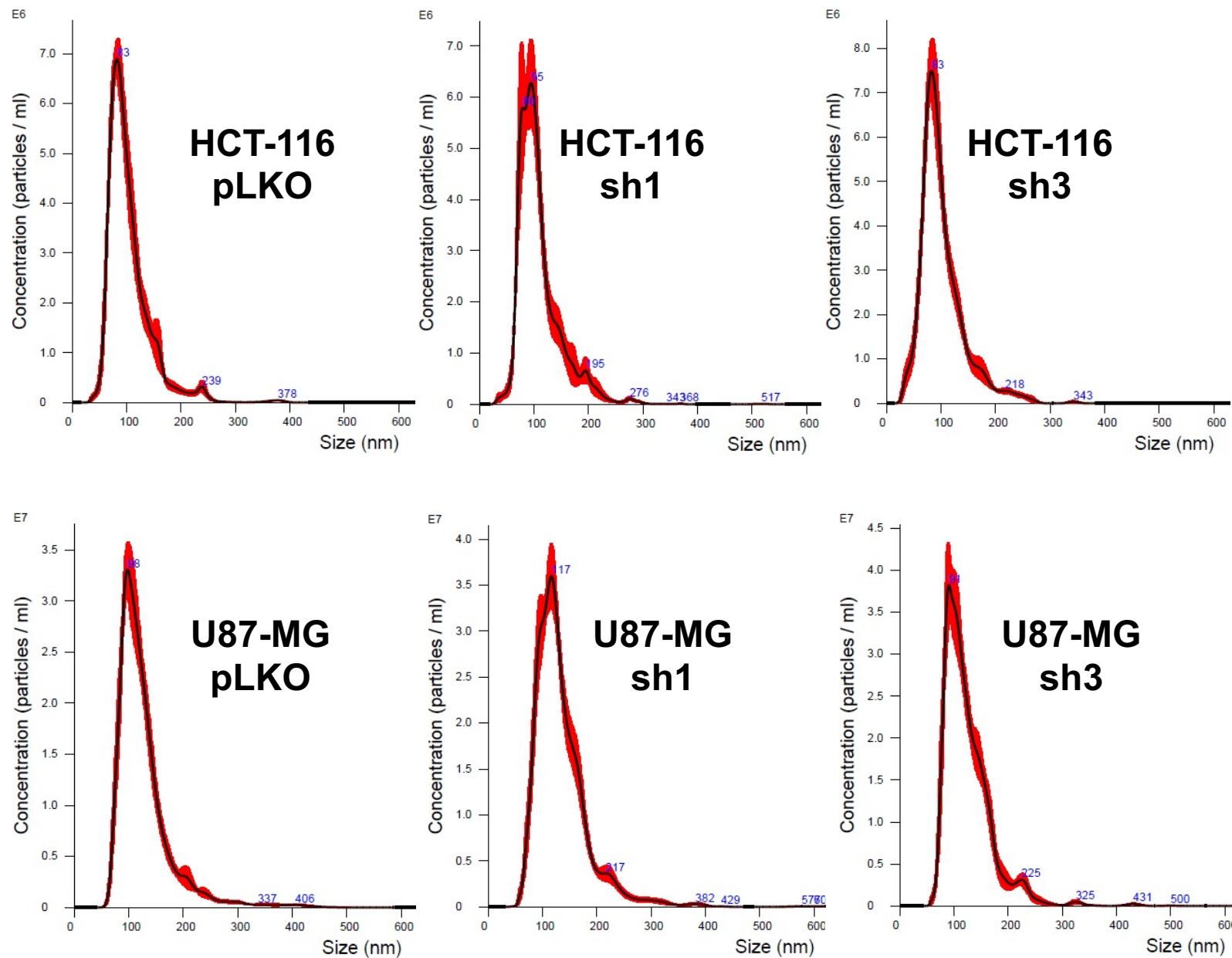

**B**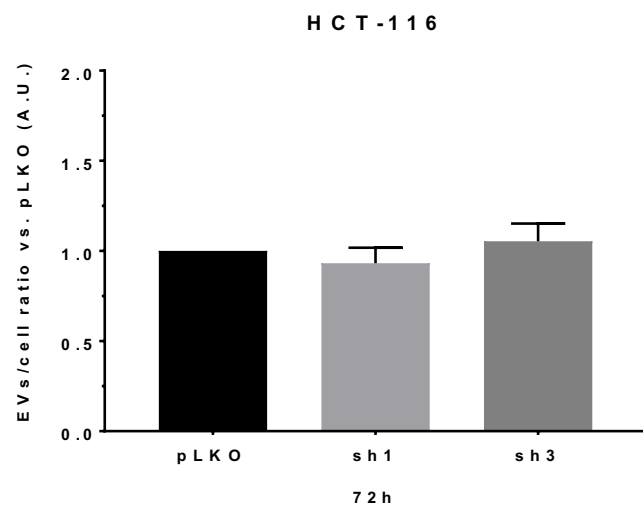**C**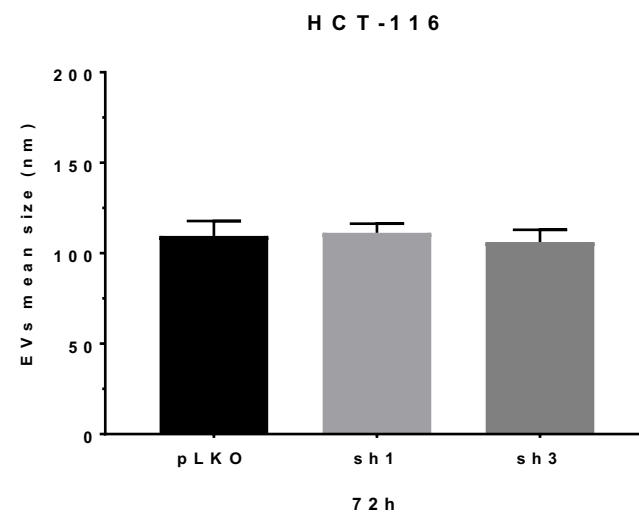**D**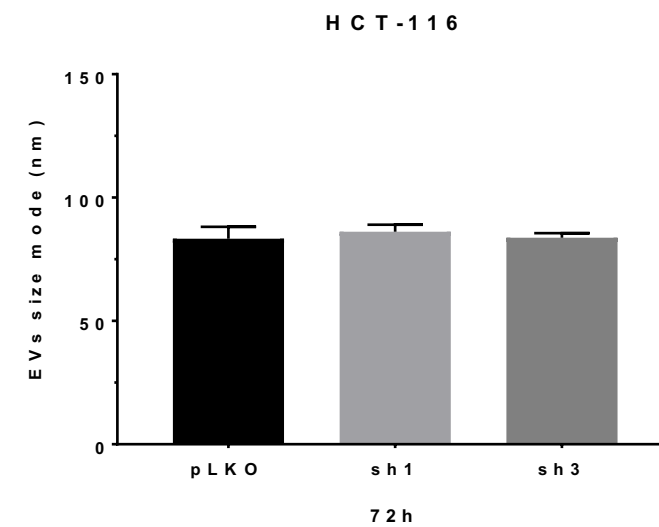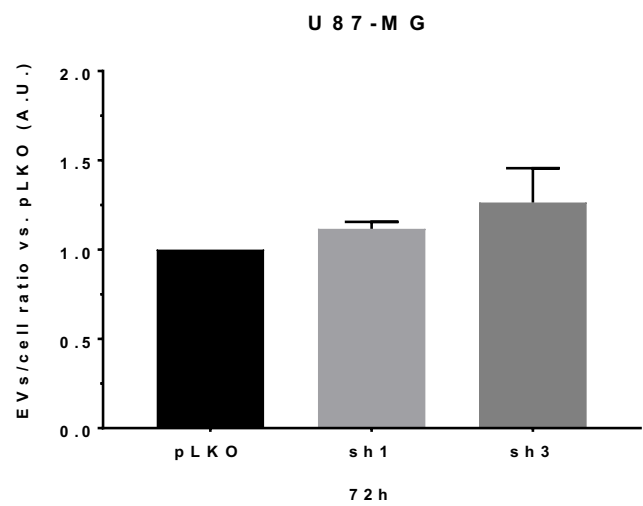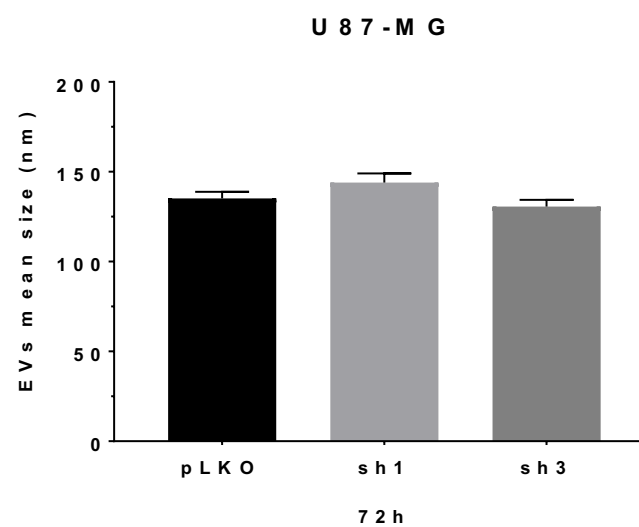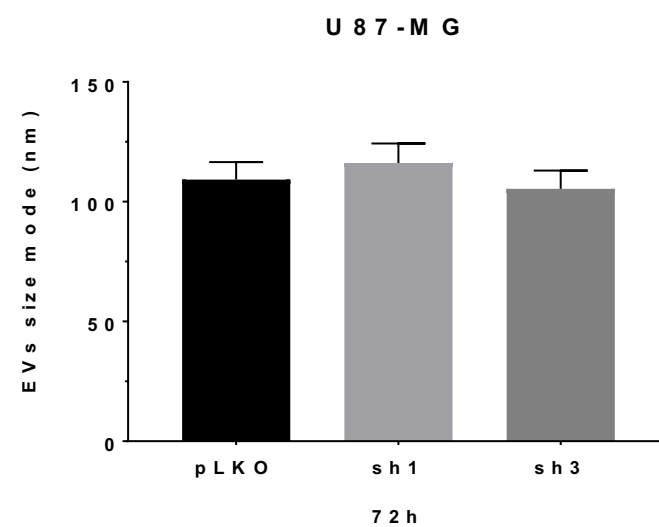

**E**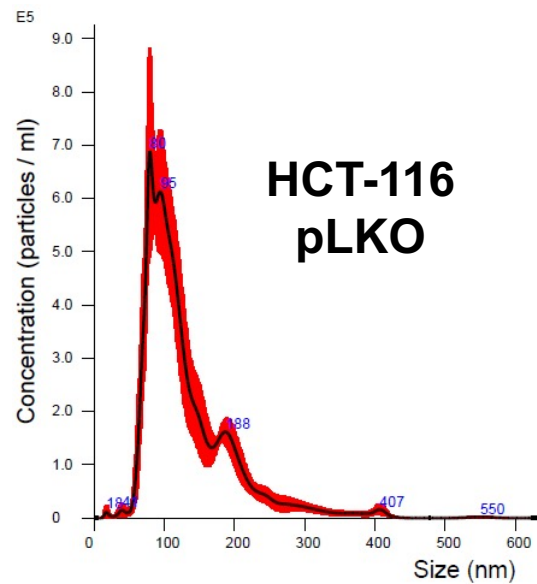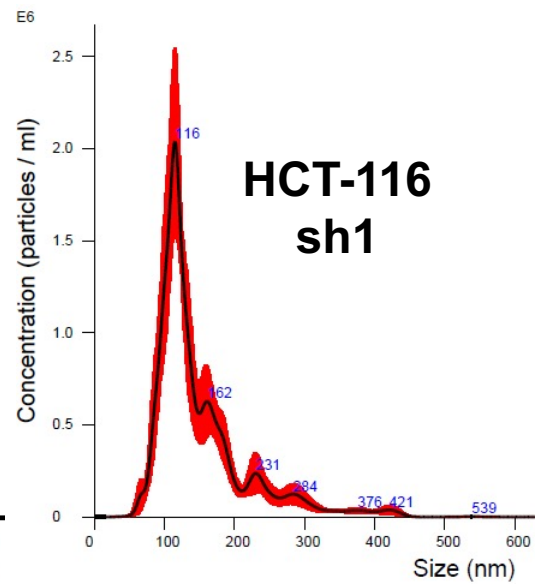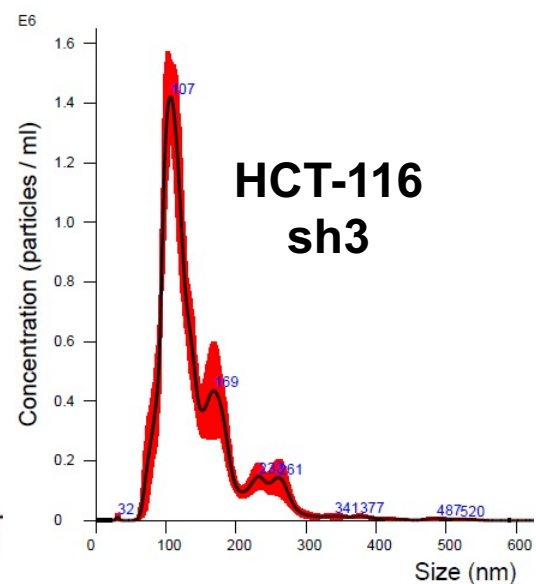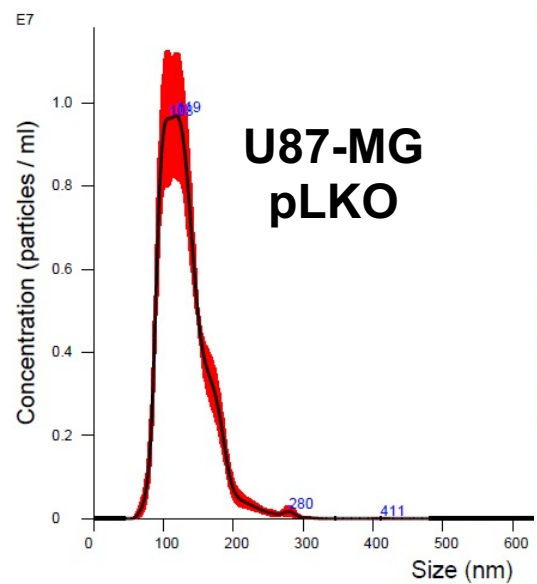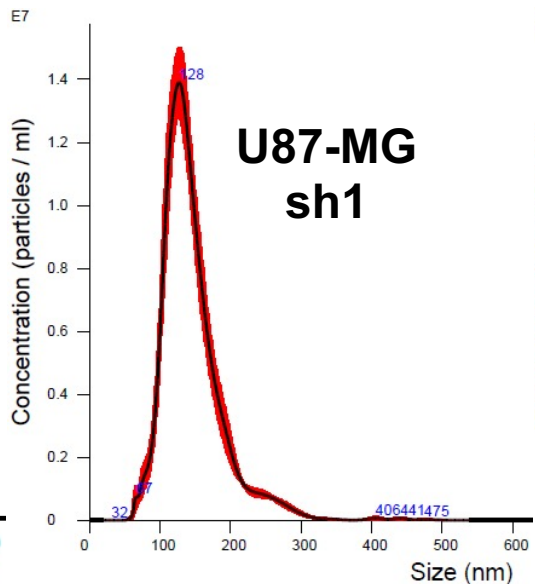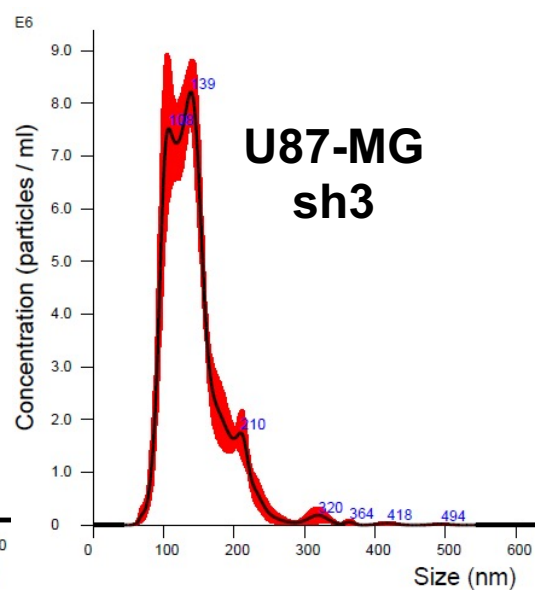

F

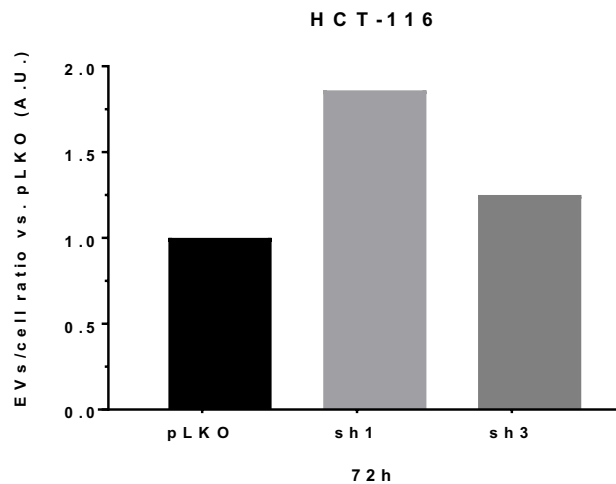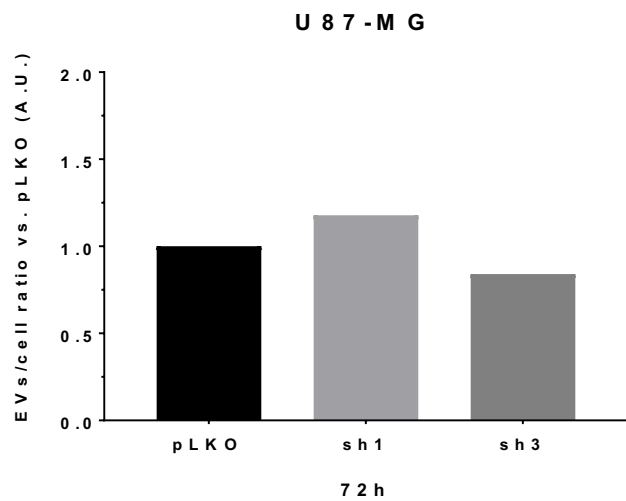

G

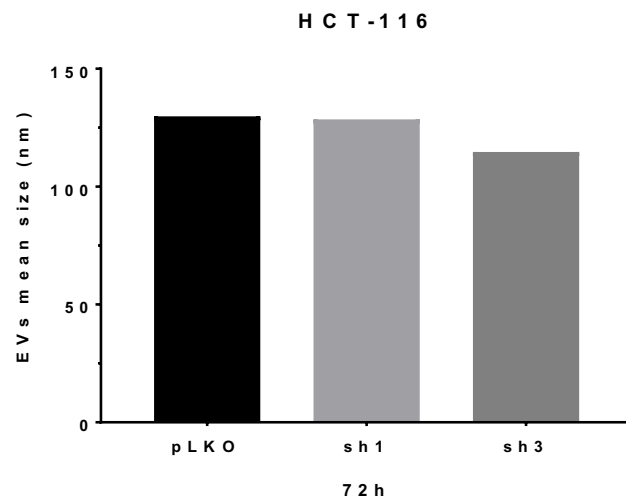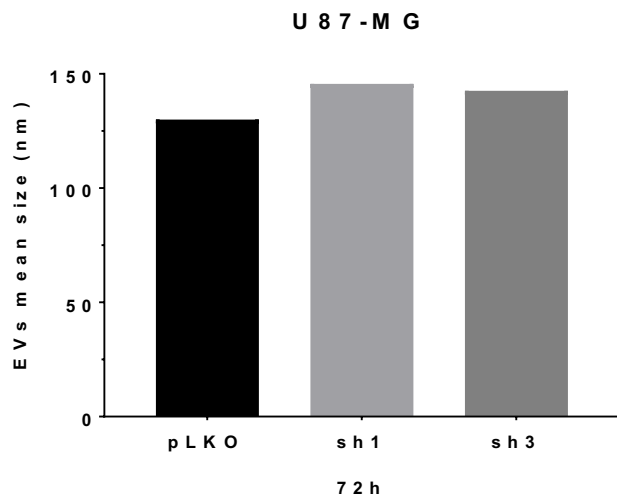

H

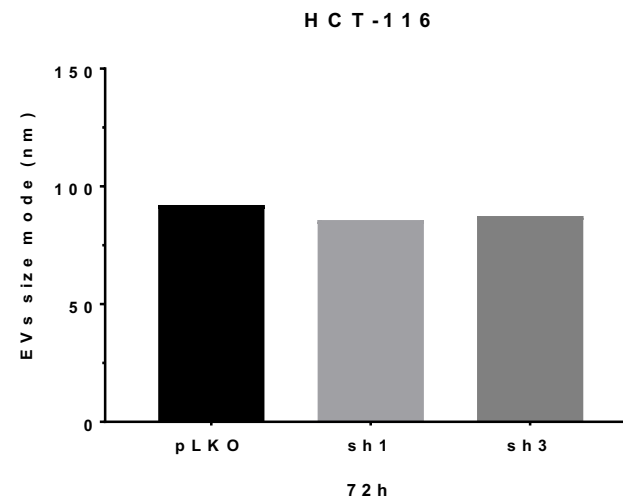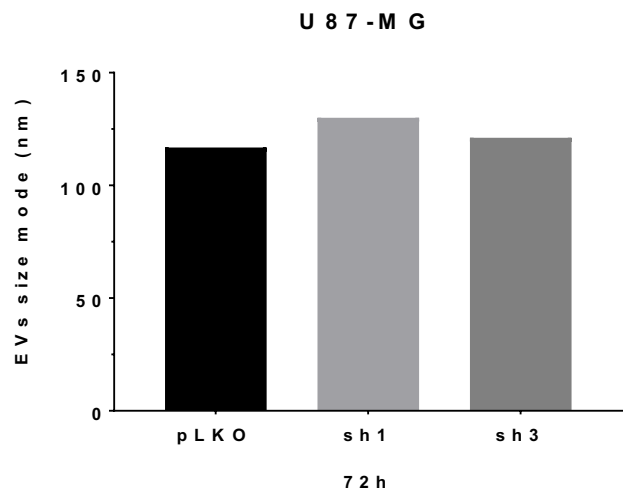

**A**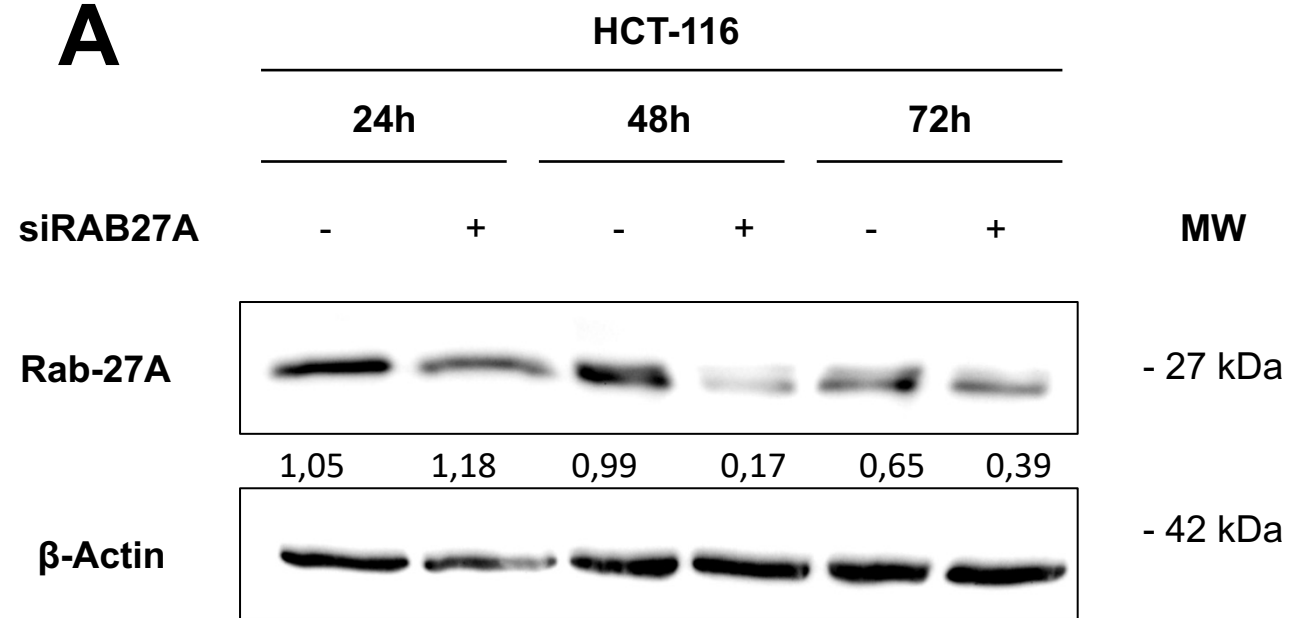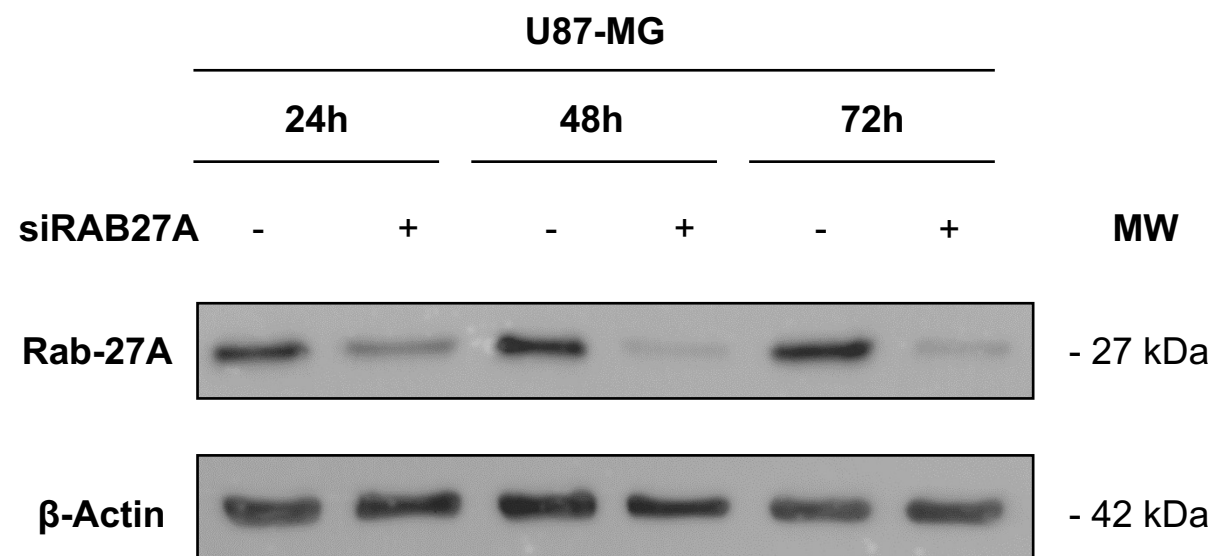

**B****HCT-116**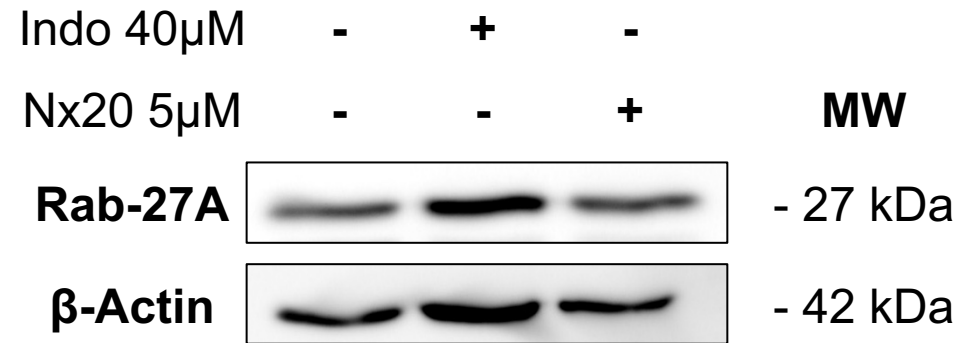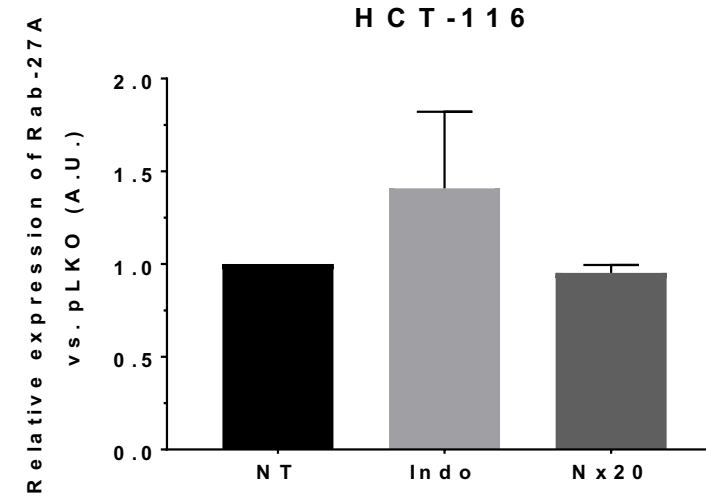**U87-pLKO**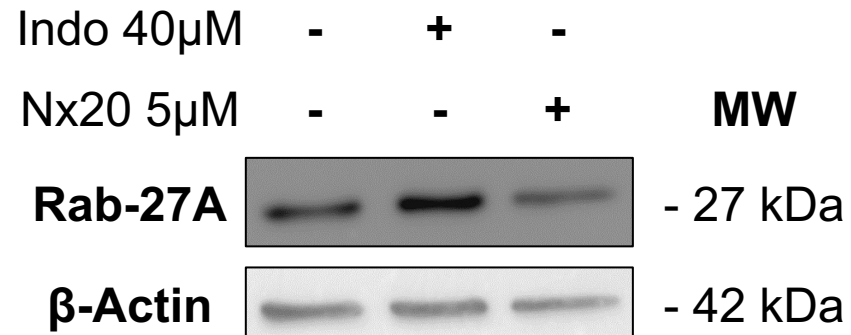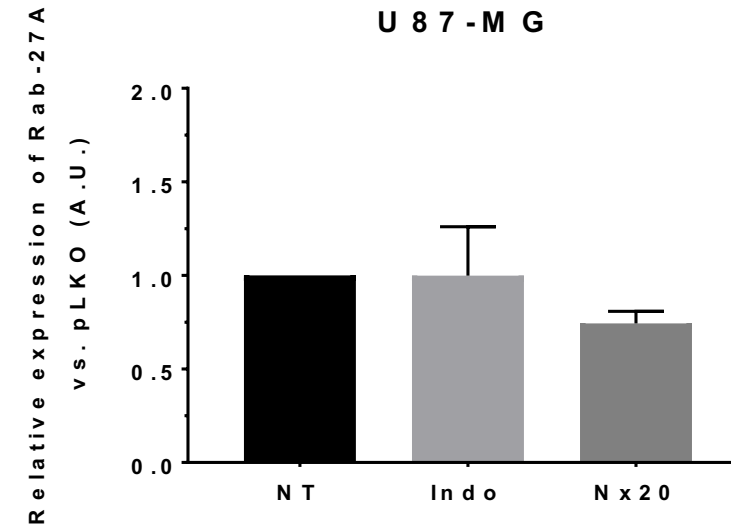

**A**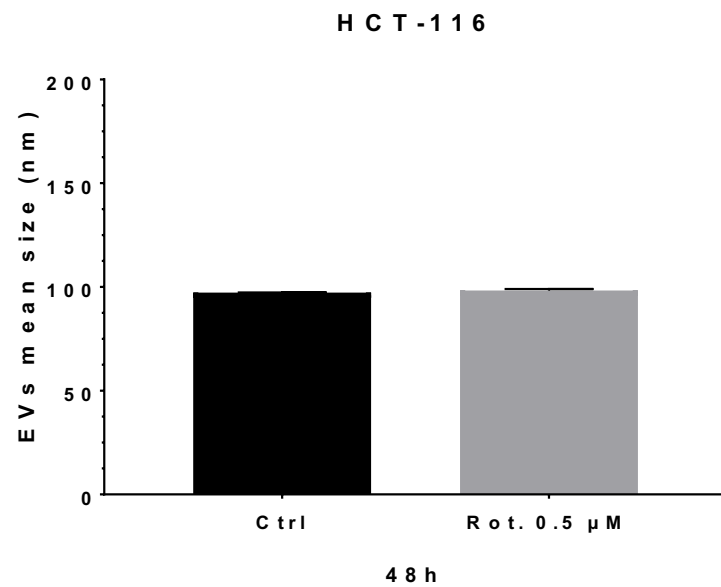**B**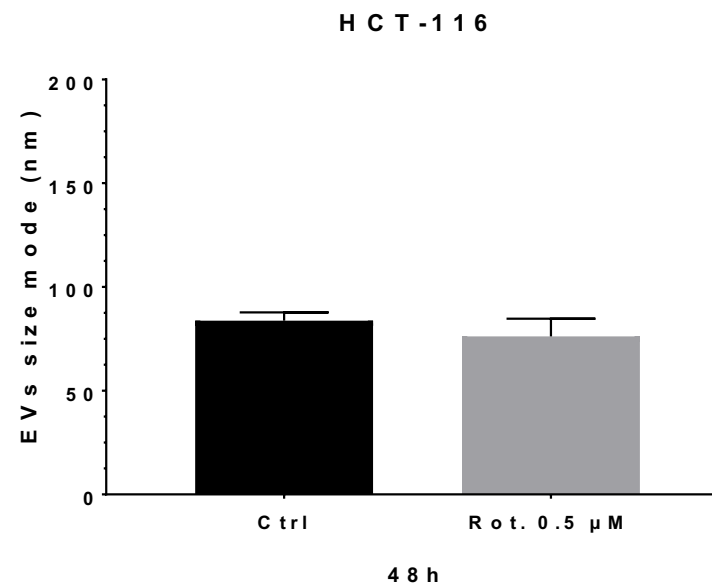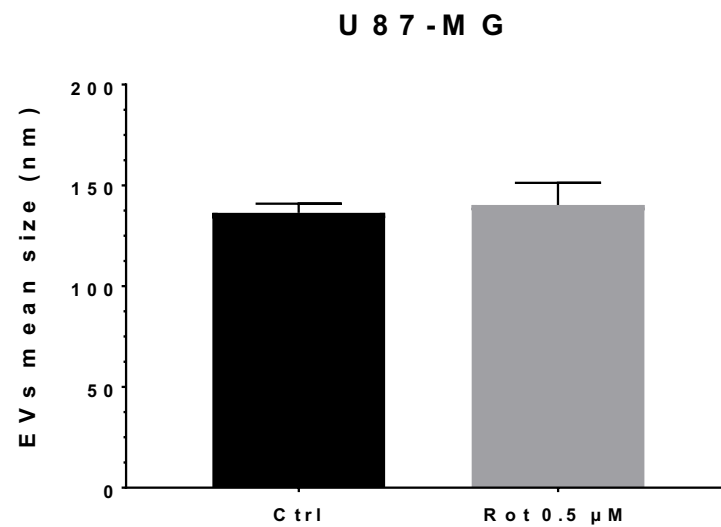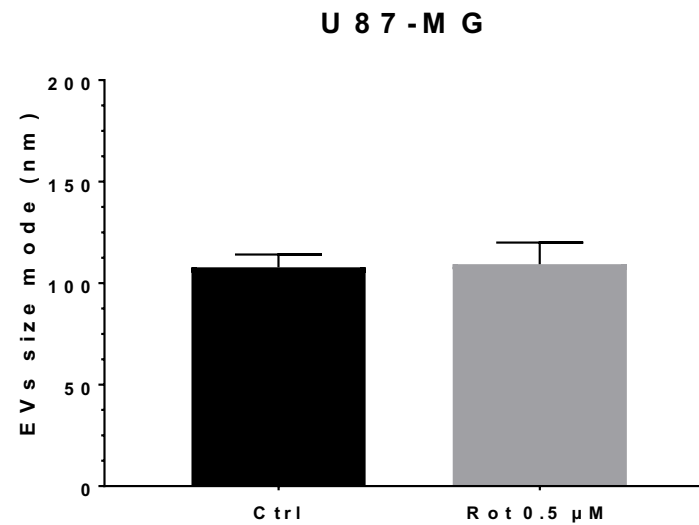

**C**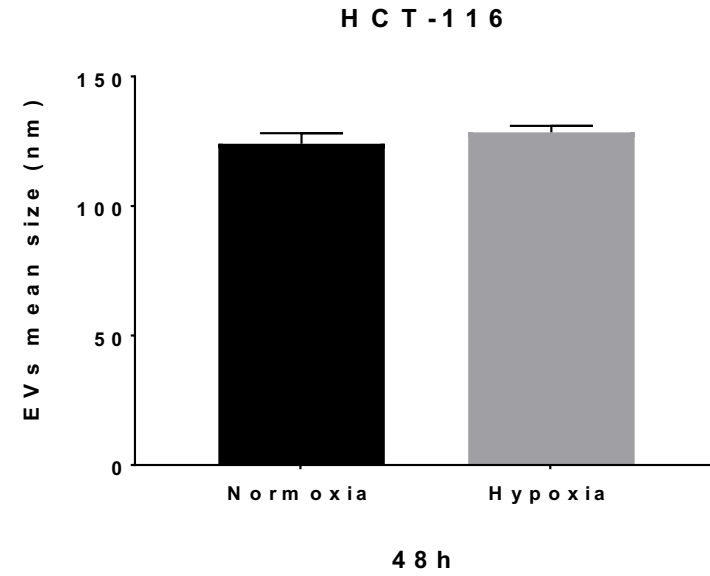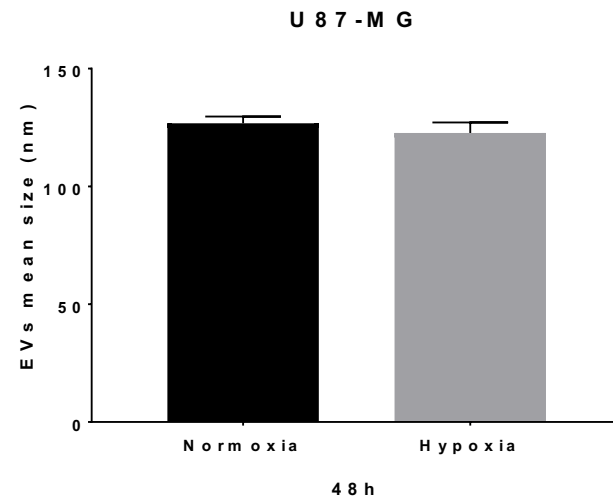**D**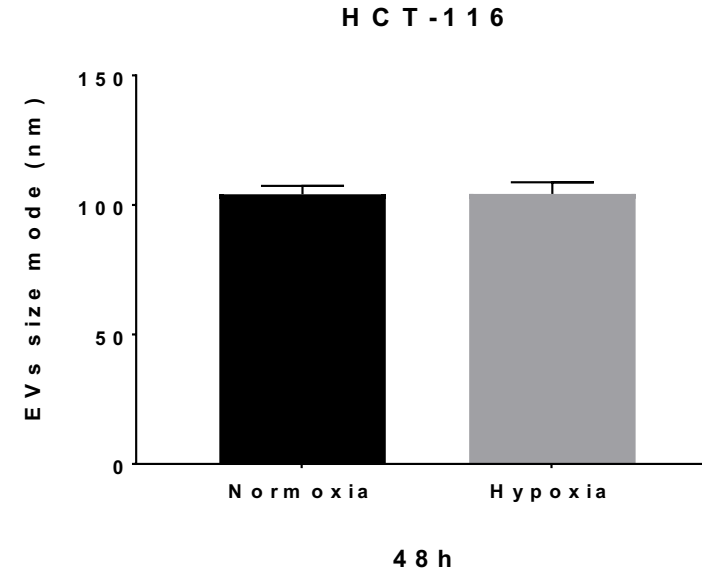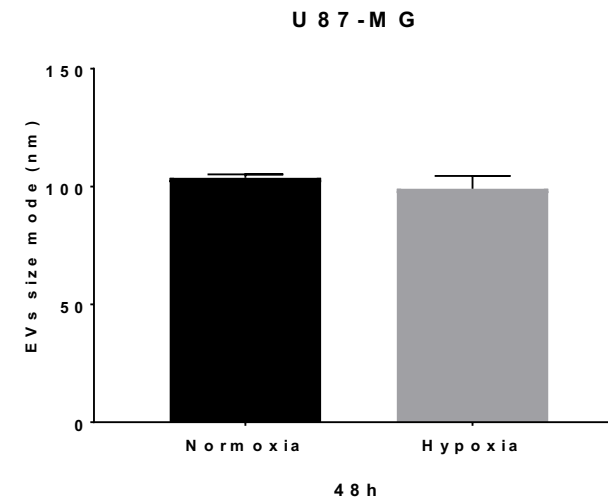

**A**

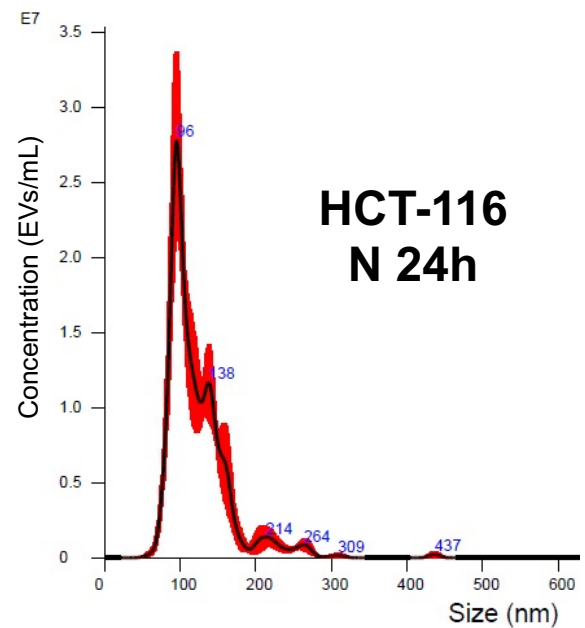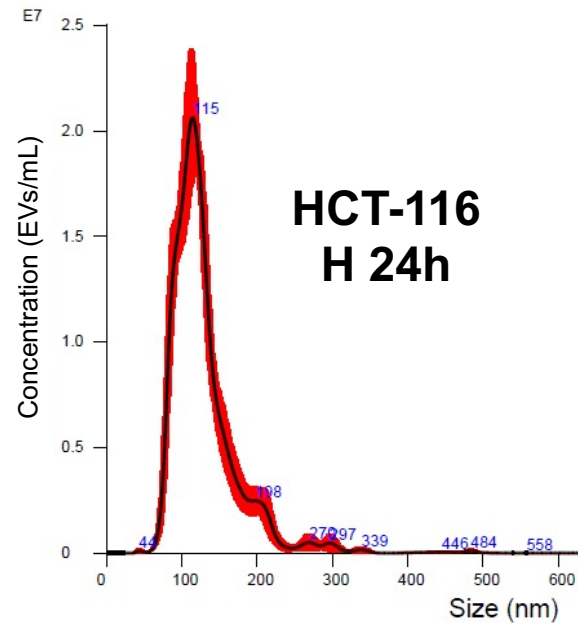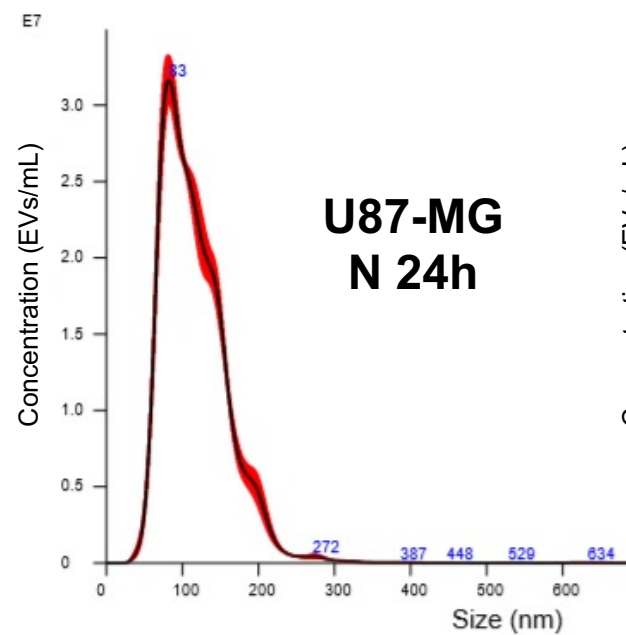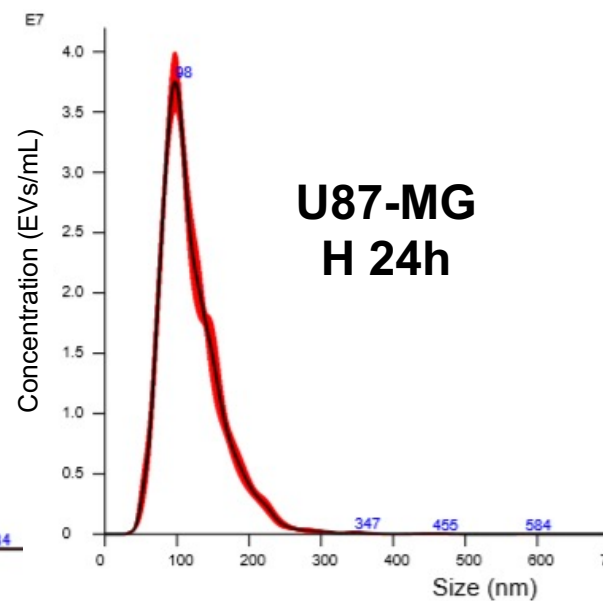

**B**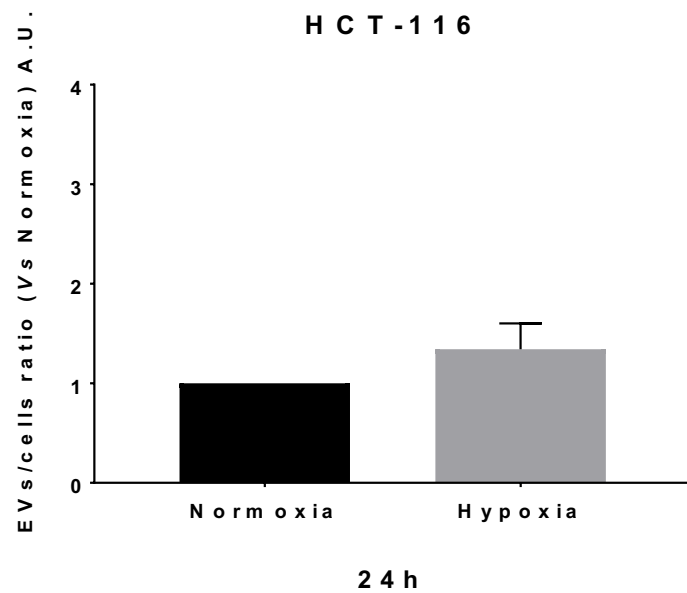**C**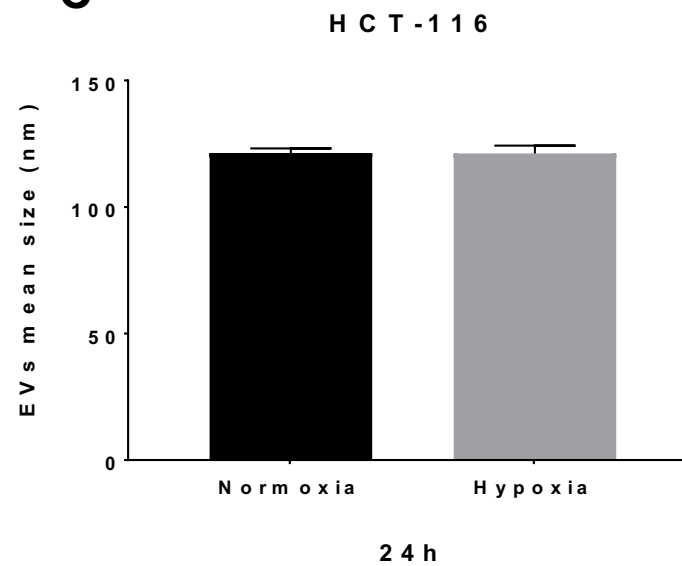**D**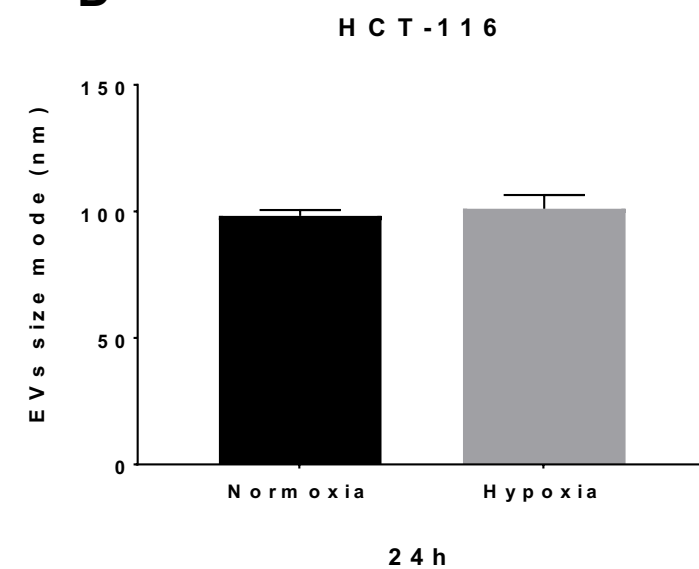**U 8 7 -M G**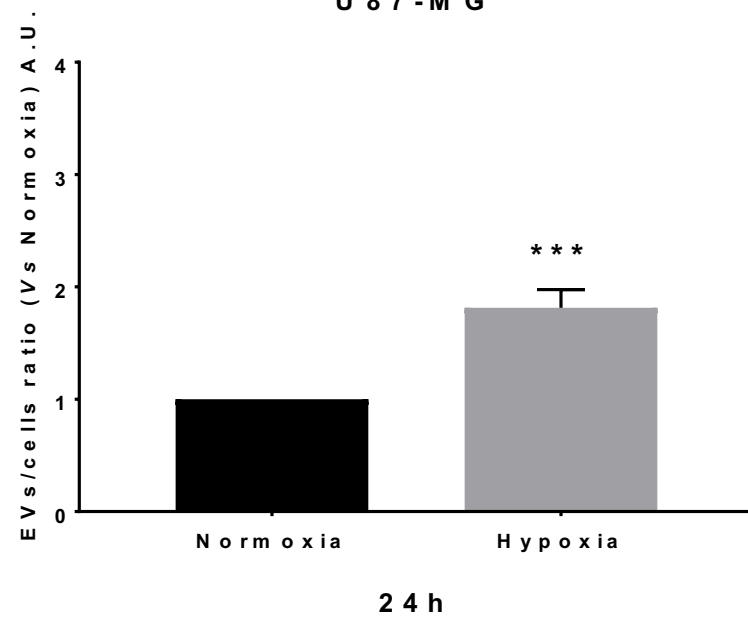**U 8 7 -M G**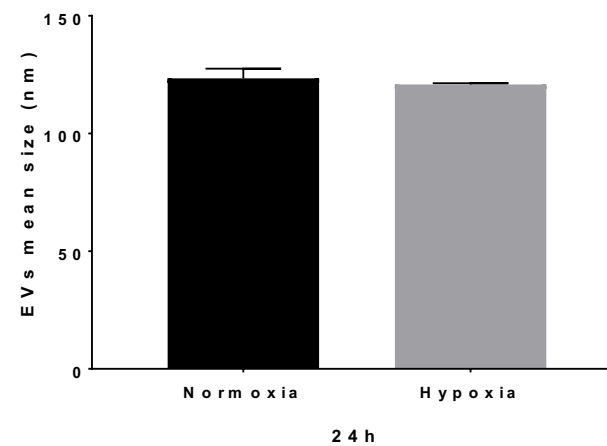**U 8 7 -M G**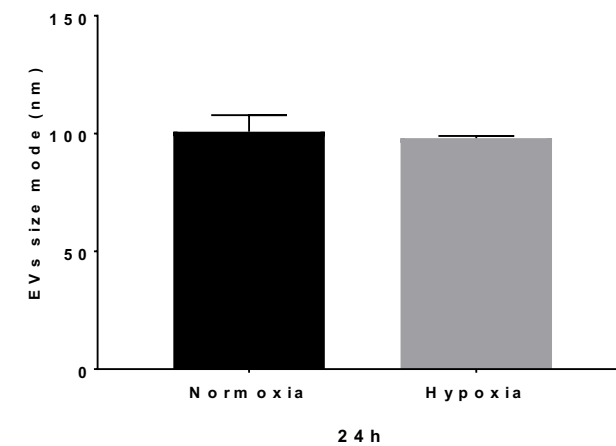

Supplement: Supplementary file 1 [file ijms-23-02310-s001.zip › ijms-1543152-supplementary-fig.pdf]
